# Yin/Yang associated differential responses to *Psoralea corylifolia* Linn. In rat models: an integrated metabolomics and transcriptomics study

**DOI:** 10.1186/s13020-023-00793-x

**Published:** 2023-08-17

**Authors:** Ming-Liang Zhang, Xu Zhao, Wei-Xia Li, Xiao-Yan Wang, Ming Niu, Hui Zhang, Yu-Long Chen, De-Xin Kong, Yuan Gao, Yu-Ming Guo, Zhao-Fang Bai, Yan-Ling Zhao, Jin-Fa Tang, Xiao-He Xiao

**Affiliations:** 1 Henan Province Engineering Laboratory for Clinical Evaluation Technology of Chinese Medicine, The First Affiliated Hospital of Henan University of Traditional Chinese Medicine, Zhengzhou, China; 2 Senior Department of Hepatology, The Fifth Medical Center of PLA General Hospital, Beijing, China; 3 Military Institute of Chinese Materia, the Fifth Medical Center of PLA General Hospital, Beijing, China; 4 Henan University of Traditional Chinese Medicine, Zhengzhou, China; 5 School of Traditional Chinese Medicine, Capital Medical University, Beijing, China; 6 College of Pharmacy, Chengdu University of Traditional Chinese Medicine, Chengdu, China; 7 Department of Pharmacy, The Fifth Medical Center of PLA General Hospital, Beijing, China

**Keywords:** *Psoraleae Fructus*, Liver injury, Predisposed individual, Metabolomics, Transcriptomics

## Abstract

**Ethnopharmacological relevance:**

*Psoralea corylifolia* Linn. (BGZ) is a commonly used traditional Chinese medicine (TCM) for the treatment of kidney-yang deficiency syndrome (Yang_syn_) with good curative effect and security. However, BGZ was also reported to induce liver injury in recent years. According to TCM theory, taking BGZ may induce a series of adverse reactions in patients with kidney-yin deficiency syndrome (Yin_syn_), which suggests that BGZ-induced liver damage may be related to its unreasonable clinical use.

**Aim of the study:**

Liver injury caused by TCM is a rare but potentially serious adverse drug reaction, and the identification of predisposed individuals for drug-induced liver injury (DILI) remains challenging. The study aimed to investigate the differential responses to BGZ in Yang_syn_ and Yin_syn_ rat models and identify the corresponding characteristic biomarkers.

**Materials and methods:**

The corresponding animal models of Yang_syn_ and Yin_syn_ were induced by hydrocortisone and thyroxine + reserpine respectively. Body weight, organ index, serum biochemistry, and Hematoxylin and Eosin (HE) staining were used to evaluate the liver toxicity effect of BGZ on rats with Yang_syn_ and Yin_syn_. Transcriptomics and metabonomics were used to screen the representative biomarkers (including metabolites and differentially expressed genes (DEGs)) changed by BGZ in Yang_syn_ and Yin_syn_ rats, respectively.

**Results:**

The level changes of liver organ index, alanine aminotransferase (ALT), and aspartate aminotransferase (AST), suggested that BGZ has liver-protective and liver-damaging effects on Yang_syn_ and Yin_syn_ rats, respectively, and the results also were confirmed by the pathological changes of liver tissue. The results showed that 102 DEGs and 27 metabolites were significantly regulated related to BGZ’s protective effect on Yang_syn_, which is mainly associated with the glycerophospholipid metabolism, arachidonic acid metabolism, pantothenate, and coenzyme A (CoA) biosynthesis pathways. While 28 DEGs and 31 metabolites, related to the pathway of pantothenate and CoA biosynthesis, were significantly regulated for the BGZ-induced liver injury in Yin_syn_. Furthermore, 4 DEGs (aldehyde dehydrogenase 1 family member B1 (Aldh1b1), solute carrier family 25 member 25 (Slc25a25), Pim-3 proto-oncogene, serine/threonine kinase (Pim3), out at first homolog (Oaf)) and 4 metabolites (phosphatidate, phosphatidylcholine, N-Acetylleucine, biliverdin) in the Yang_syn_ group and 1 DEG [galectin 5 (Lgals5)] and 1 metabolite (5-amino-1-(5-phospho-D-ribosyl)imidazole-4-carboxylate) in Yin_syn_ group were significantly correlated to the ALT and AST levels of BGZ treated and untreated groups (receiver operating characteristic (ROC) ≥ 0.9).

**Conclusions:**

Yin_syn_ and Yang_syn_ are the predisposed syndromes for BGZ to exert liver damage and liver protection respectively, which are mainly related to the regulation of amino acid metabolism, lipid metabolism, energy metabolism, and metabolism of cofactors and vitamins. The results further suggest that attention should be paid to the selection of predisposed populations when using drugs related to the regulation of energy metabolism, and the Yin_syn_/Yang_syn_ animal models based on the theory of TCM syndromes may be a feasible method for identifying the susceptible population to receive TCM.

**Supplementary Information:**

The online version contains supplementary material available at 10.1186/s13020-023-00793-x.

## Introduction

Drug-induced liver injury (DILI) refers to one of the most common and serious adverse drug reactions induced by various prescription or non-prescription chemical drugs, biological agents, traditional Chinese medicine (TCM), natural medicine, health care products, dietary supplements and their metabolites and even excipients, which can lead to acute liver failure or even death [1]. Due to the lack of drug-specific biomarkers, the diagnosis of DILI mainly depends on clinical judgment and exclusion of other liver diseases, and then through the causal relationship assessment to determine the degree of association between liver injury and suspected drugs [2]. Thus, the lack of evidence-based and reliable diagnostic tools has always been one of the prominent problems in the clinical diagnosis, treatment, and research of DILI. However, the proportion of DILI caused by traditional Chinese herbs and dietary supplements has increased year by year [3, 4]. This is particularly noticeable for non-toxic TCM such as *Polygonum multiflorum Thunb.*[5] and *Psoralea corylifolia* Linn (BGZ) [6], which have been used for thousands of years in China. This phenomenon is causing great confusion for doctors and patients. Thus, it is particularly critical to find the factors that induce DILI (especially DILI caused by TCM) and the specific biomarkers that characterize DILI.

DILI usually involves two factors, the “drug” and the “host” [7]. For drug factors, Chen et al. constructed a “role-of-two” model [8] and modified the version used [9]. The model enhanced the ability to predict whether a drug will cause DILI, but it was unable to fully explain individual differences in drug usage. Therefore, it cannot be used to predict individuals who are predisposed to DILI. Host factors are also important in understanding the susceptibility to DILI. Host factors that are generally recognized by modern research include hosts who carry specific HLA genes or the immune homeostasis of the host [9]. As reported in recent years, liver damages induced by *Polygonum multiflorum* Thunb., Green Tea, and Kampo products (Japanese traditional medicines), etc. are closely related to HLA-B*35:01 [10–12], while liver damage induced by flucloxacillin, pazopanib, abacavir, etc. are closely related to HLA-B*57:01 [13–17]. Screening susceptible genes and identifying susceptible populations are important methods to prevent the occurrence of liver injury caused by TCM. However, up to now, most of the hepatotoxicity of TCM has not been found in susceptible genes, and even if the susceptible gene is found, it does not necessarily represent that the person carrying the susceptible gene will have a specific liver injury when taking the TCM.

Actually, the identification of susceptible genes or populations is similar to the “*therapy with syndrome differentiation”* of TCM, both of which are based on identifying drug-susceptible populations for rational medication use. However, for TCM theory, in addition to the correct understanding of medicine, it is also necessary to correctly identify TCM syndromes that summarize the pathological changes of the body at a certain stage of disease development based on TCM theory. TCM treatment requires “*therapy with syndrome differentiation*”. Symptomatic treatment can result in a better therapeutic effect, otherwise, it can aggravate the disease process and induce new diseases. Therefore, it may be feasible to explore the predisposition of individuals to TCM liver injury based on TCM syndrome theory which may provide a reference for other similar studies on the hepatotoxicity of TCM.

BGZ, the dried mature fruit of the leguminous plant *Psoralea corylifolia* Linn, is widely used in Asia and has been used for many years in China to treat symptoms such as impotence, nocturnal emission, enuresis, frequent urination, cold aching in the lower back and knees, kidney deficiency, and premature ejaculation [18]. BGZ has definite pharmacological effects, such as anti-tumor, anti-oxidation, antibacterial, anti-inflammatory, anti-depression, estrogen level regulation, bone growth promotion, nerve protection, and influence on the liver [19]. Its active ingredients, psoralen, and 5-methoxypsoralen, have become commonly used in the clinical treatment of vitiligo and psoriasis [20, 21]. In addition, BGZ and its related compounds are also commonly used in health food and dietary supplements. However, in recent years, BGZ and its related compound preparations Zhuanggu Guanjie Pill and Xianling Gubao Capsules were reported to induce DILI in China [6, 22–24]. The BGZ-induced liver injury also occurred in South Korea [25].

Although some studies have confirmed that BGZ and some of its components have hepatotoxicity [6, 26, 27], these studies only focused on the drug itself and did not consider the host factors that induced DILI. Accordingly, by integrating the basic disease characteristics of the population treated with BGZ, our previous study found that most BGZ-induced DILI patients had osteoporosis, psoriasis, osteoarthritis, and other basic diseases related to immune activation [28], and such diseases were found to be closely related to the imbalance of kidney-yang deficiency syndrome (Yang_syn_) and kidney-yin deficiency syndrome (Yin_syn_) according to TCM diagnosis [29–31]. The ancient TCM medicine book “*Lei Gong Concocting (Paozhi) Theory*” also recorded during the Northern and Southern Dynasties in China that people with “asthenic yin causing excessive pyrexia” should avoid taking BGZ. Thus, the BGZ-induced DILI may be related to its inappropriate symptomatic treatment. However, whether it has a causal relationship with the imbalance of Yang_syn_ and Yin_syn_ in the body remains unclear.

According to the diagnostic criteria of TCM syndromes [32], patients with Yang_syn_ generally have low body temperature, loose stools, and less drinking and urine, while patients with Yin_syn_ generally have high body temperature, dry stool, polydipsia, and polyuria. In addition, some studies have found that the expression of cAMP in patients with Yang_syn_ decreased [33, 34], while the expression of cAMP in patients with Yin_syn_ increased [34, 35]. Accordingly, continuously administered high doses of exogenous glucocorticoid (GC) (e.g., hydrocortisone) [36] or thyroxine + reserpine [37] in rats were used to build the models of Yang_syn_ and Yin_syn_, respectively. The changes in behavioral signs and cAMP levels in the above animal models are similar to the clinical patients with the corresponding syndromes and have been generally recognized and adopted by experts in the related fields of TCM. With a good description of the changes in the metabolic characteristics of endogenous metabolites and the differential expression changes of a series of functional genes in organisms, metabolomics and transcriptomics have been successfully applied to screen various diseases and their metabolic or gene profile changes in drug intervention [38, 39]. This study constructed Yang_syn_ and Yin_syn_ animal models to evaluate whether the liver damage induced by BGZ is related to its non-symptomatic use and the possible susceptible individual characteristics.

## Experiment

### Chemicals and reagents

Hydrocortisone succinate sodium was provided by Tianjin Biochemical Pharmaceutical Co., Ltd. (Tianjin, China). Thyroxine and reserpine were provided by Shanghai MACKLIN Technology Co., Ltd. (Shanghai, China). Alanine aminotransferase (ALT), aspartate aminotransferase (AST), serum creatinine (Scr), lactate dehydrogenase (LDH), and Na^+^-K^+^-ATP_ase_ tests were purchased from Jiancheng Biological Technology, Co., Ltd. (Nanjing, China). Cyclic adenosine monophosphate (cAMP) was purchased from Mlbio Biotechnology Co., Ltd. (Shanghai, China).

### BGZ Preparation

BGZ was purchased from the Anhui Puren Herbal Pieces Co., Ltd. (Anhui, China) and authenticated by Professor Xiaohe Xiao of the Institute of Hepatology, Fifth Medical Center, PLA General Hospital (Beijing, China). In addition, BGZ was crushed and filtered through 200 mesh sieves and then suspended in 0.5% sodium carboxymethyl cellulose (CMC-Na) for administration.

### Animal maintenance and treatment

Male Sprague–Dawley rats (180–200 g) were obtained from the SPF Biotechnology Co. Ltd. (License No. SCXK20190010, Beijing, China), and housed in the Laboratory Animal Center of the Fifth Medical Center, Chinese PLA General Hospital (animal ethics committee approval No. YFYDW2020017). All rats were raised under specific pathogen-free conditions under a 12 h light/dark cycle, with free access to adequate food and water. All animals were fed adaptively for 1 week before starting the experiments.

The rats were randomly divided into six separate groups (N = 8) as follows: Control group (CON), BGZ group (BGZ), kidney-yang deficiency syndrome group (Yang_syn_), kidney-yang deficiency syndrome-treated with BGZ group (Yang_syn_+BGZ), kidney-yin deficiency syndrome group (Yin_syn_); kidney-yin deficiency syndrome-treated with BGZ group (Yin_syn_+BGZ). Rats in the BGZ-related treatment groups were consecutively administered intragastrically with BGZ suspension for 21 consecutive days, while the CON, Yang_syn_, and Yin_syn_ groups received the same volume of 0.50% CMC-Na solution for the same amount of time. The changes in body weight were recorded before the end of the experiment. From the 8th day of administration, the Yang_syn_ and Yang_syn_+BGZ group rats were given 25 mg/kg hydrocortisone subcutaneously once a day for 14 consecutive days to prepare the Yang_syn_ model [36]. From the 15th day of administration, the Yin_syn_ and Yin_syn_+BGZ group rats were given thyroxine (16 mg/mL) and reserpine (1 mg/mL) at the dosage of 0.5 mL/100 g via gastric perfusion once a day for 7 consecutive days to prepare the Yin_syn_ model [37]. The CON and the BGZ groups were given the same volume of 0.50% CMC-Na solution.

### Urine volume, water intake, anal temperature, fecal water content changes

According to the diagnostic criteria of TCM syndromes [32], patients with Yang_syn_ generally have low body temperature, loose stools, less drinking, and less urine, while patients with Yin_syn_ generally have high body temperature, dry stool, polydipsia, and polyuria. Thus, the anal temperature, fecal water content, 24 h drinking water volume, and 24 h urine volume before the last administration were selected as the criteria to evaluate whether the models of Yang_syn_ and Yin_syn_ were successfully constructed as the previous reports [40, 41].

### Blood collection, organ index, and tissue preparation

After the experiment, all animals were anesthetized with 2% pentobarbital sodium. Blood samples with and without anticoagulants were collected, and the liver, kidney, adrenal gland, testis, spleen, brain, lung, and heart were weighed immediately after sacrifice to calculate the organ index. Partial liver tissue was collected for histological examination and the remaining liver was quickly frozen with liquid nitrogen and stored at − 80 °C until needed.

### Serum biochemistry and histopathological analysis

After centrifugation (3500 rpm, 10 min, 4 °C), serum biochemistry of ALT, AST, Scr, LDH, Na^+^-K^+^-ATP_ase,_ and cAMP were determined according to the microplate assay kit instructions, the left hepatic lobe was fixed with 4% paraformaldehyde for 48 h, embedded in wax, and sectioned at approximately 5 μm for Hematoxylin and Eosin (HE) pathological staining analysis.

### RNA sequence analysis and data processing

Total RNA was extracted from liver tissues using Trizol reagent (Invitrogen, USA) according to the manufacturer’s protocol, and genomic DNA was removed using DNase I (TaKara). RNA quality was assessed with a Bioanalyzer 2100 (Agilent) and measured with a NanoDrop 2000 spectrophotometer. Only a high-quality RNA sample (OD_260/280_ = 1.8 ~ 2.2, OD_260/230_ ≥ 2.0, RNA integrity number (RIN) ≥ 6.5, 28 S:18 S ≥ 1.0, > 1 µg) was used to construct the sequencing library. RNA-seq transcriptome libraries were prepared using a TruSeqTMRNA sample preparation kit from Illumina (San Diego, CA) and sequenced with the Illumina HiSeq xten/NovaSeq 6000 sequencer under standard protocols. All samples had a Q30 (bases of Q ≥ 30 /all bases of sequencing) of > 91%. Sequence readers were trimmed and quality controlled using SeqPrep (https://github.com/jstjohn/SeqPrep) and Sickle (https://github.com/najoshi/sickle) with default parameters and aligned to the reference genome through the orientation mode using hierarchical indexing for spliced alignment of transcripts 2 (HISAT2) [42]. Messenger RNA levels were quantified using RNA-Seq by Expectation-Maximization (RSEM) (http://deweylab.biostat.wisc.edu/rsem/) [43]. Differentially expressed genes (DEGs) were identified as those with fold change (FC) ≥ 1.5, (FC) ≤ 0.67, and *Padjust < 0.05* (DESeq2) [44]. Furthermore, Gene Ontology (GO, http://www.geneontology.org) functional enrichment and Kyoto Encyclopedia of Genes and Genomes (KEGG, http://www.genome.jp/kegg/) pathway analysis carried out by Goatools (https://github.com/tanghaibao/Goatools) and KOBAS (http://kobas.cbi.pku.edu.cn/home.do) were performed to determine significant GO terms and pathways associated with the DEGs [45]. Pathways with *Padjust < 0.05* were considered potential target pathways.

### Reverse transcription-quantitative polymerase chain reaction (RT-qPCR)

Liver tissue RNA was extracted by the Tissue RNA Purification Kit Plus (RN002plus, ES Science, China) and reverse-transcribed into cDNA using the Fast All-in-One RT Kit (RT001, ES Science, China) according to the manufacturer’s instructions. The qPCR of aldehyde dehydrogenase 1 family member B1 (Aldh1b1), galectin 5 (Lgals5), solute carrier family 25 member 25 (Slc25a25), Pim-3 proto-oncogene, serine/threonine kinase (Pim3), and out at first (Oaf) were quantified by the SYBR Green PCR master mix (RN002plus, ES Science, China) with the QuantStudio 6 Flex PCR System (Applied Biosystems, USA). The amplification parameters were set according to the standard protocol. Primer sequences used in this study are shown in Additional file 1: Table S1. Relative gene expression was calculated using the 2^−△△Ct^ method 2^−∆∆Ct^ [46].

### Liver sample processing

The liver was homogenized with normal saline at 1:1 (1 g:1 mL) using a homogenizer. The 300 uL homogenized sample and 900 uL methanol were mixed and vortexed for 30 s. The supernatant was centrifuged at 12,000 r/min for 10 min and concentrated to dry using a vacuum centrifugal concentrator. Then 100 uL methanol was added for redissolving and centrifuged at 13,000 r/min for 10 min. Finally, a 4 uL supernatant was taken out for UPLC-QTOF/MS (Waters, Manchester, UK) detection.

### UPLC-QTOF/MS analysis and data processing

Metabolic profiling analysis of the biofluids was performed using the Waters Xevo G2-XS QTOF/MS (Waters, Manchester, UK). An analytical Acquity UPLC HSS T3 C18 column (temperature 30 °C) was injected with 4 µL aliquots of each sample. For positive electrospray ionization source (ESI+) and negative electrospray ionization source (ESI-) analysis, samples were isolated using a 30 min linear gradient of solvent A (water spiked with 0.1% formic acid) and solvent B (acetonitrile spiked with 0.1% formic acid) as mobile phases. The flow rate was fixed at 0.30 mL/min. For each sample, 10 µL was drawn as a quality control sample to ensure that the system was stable and the analyses were repeatable. Every 20th sample was injected with the control sample and subsequently analyzed. Masslynx software (v4.1, Waters Corp.) and Progenesis QI (v. 2.4, Waters Technologies, UK) were used for identifying the original mass spectral data and normalizing the total ion intensity of each chromatogram to acquire a data matrix containing the m/z value, retention time (RT), and normalized peak area. SIMCA-P 14.1 software (Umetrics, Umea, Sweden) was used for principal component analysis (PCA) and orthogonal partial least-squares discriminant analysis (OPLS-DA). The PCA score chart was used to show the natural interrelation of observation results. Variable importance in the projection (VIP) ≥ 1 and *P < 0.05* were selected as potential metabolites. Online metabolic databases including the Human Metabolome Database (HMDB) (http://www.hmdb.ca/) and Kyoto Encyclopedia of Genes and Genomes (KEGG) (https://www.genome.jp/kegg/) were combined with exact masses and secondary ion mass spectrometry of the metabolites acquired through Progenesis QI to identify the differential metabolites.

### Integrated analysis

Cytoscape 3.8.0 (https://js.cytoscape.org/) is a visualization tool for exploring biomedical networks composed of compounds, genes, and other types of interactions [47]. MetScape (http://MetScape.ncibi.org) is a plugin of Cytoscape that allows users to construct and analyze gene and compound networks, and identify and visualize enriched pathways changes in expression profiling data and metabolite data [48]. The data obtained for differentially abundant metabolites and DEGs from rats in the Yang_syn_/Yin_syn_ and corresponding BGZ-treated groups were imported into Metscape to obtain a global understanding of gene and metabolic changes to assess the underlying mechanisms of Yin_syn_/Yang_syn_-associated differential responses to BGZ in rat models, respectively.

### Statistical analysis

Statistical analysis was performed with Prism 8.0 (GraphPad Software, San Diego, CA). All results are expressed as mean ± SD. A one-way analysis of variance (ANOVA) was used to statistically analyze the multiple-group analysis. The student’s t-test was used to statistically analyze the receiver operating characteristic (ROC) curve analysis. The significance threshold was set at *P < 0.05*.

## Results

### Effect of BGZ treatment on the changes in body weight, anal temperature, water intake, urine volume, fecal water content, and cAMP in Yin_syn_ and Yang_syn_ model rats

**Fig. 1:**
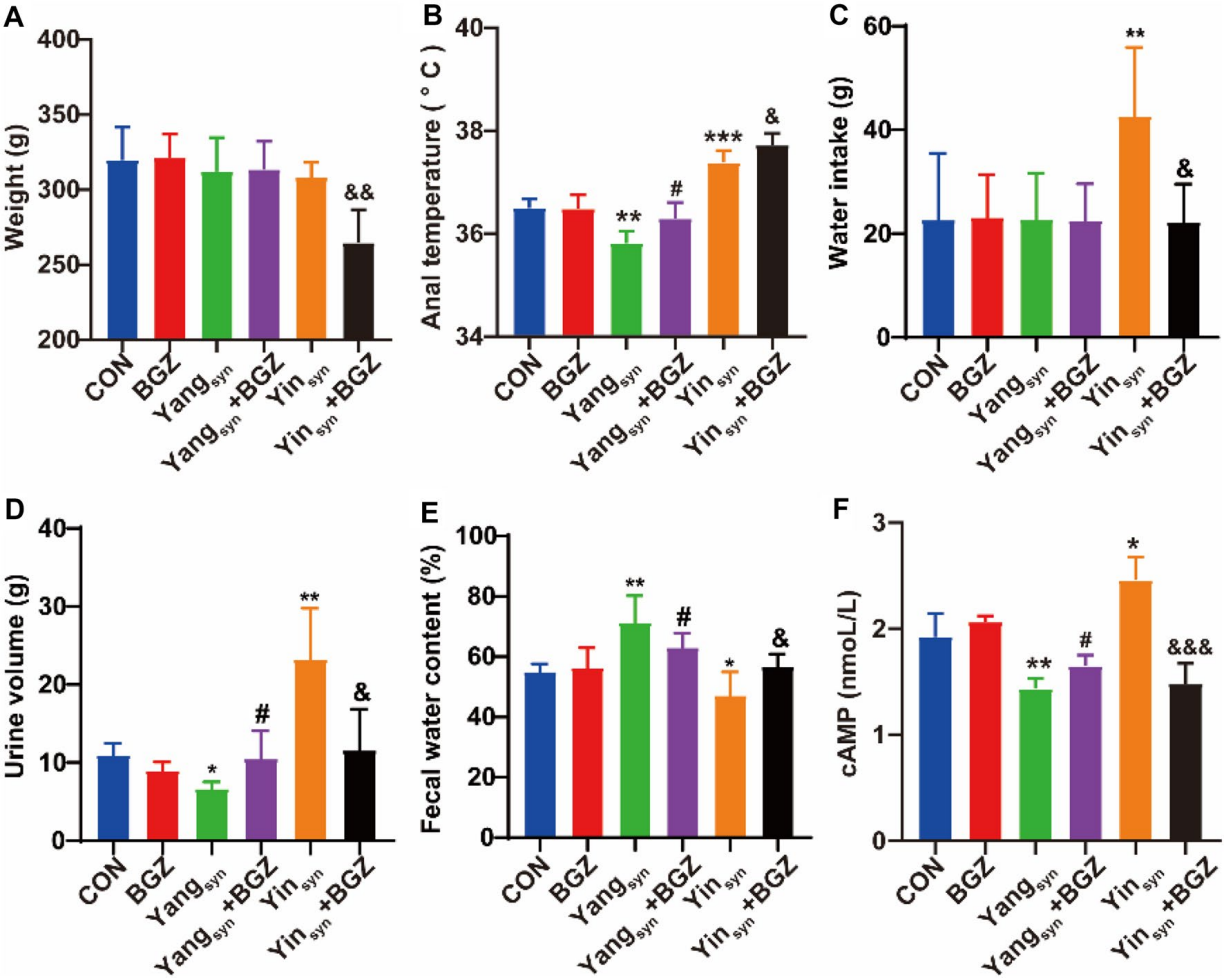
Effect of BGZ treatment on changes in body weight, anal temperature, water intake, urine volume, fecal water content, and cAMP (N = 6 ~ 8). Weight (**A**), Anal temperature (**B**), Water intake (**C**), Urine volume (**D**), Fecal water content (**E**), and cAMP (**F**). *P < 0.05, **P < 0.01, ***P < 0.001, compared with CON group; ^*#*^*P < 0.05*, compared with Yang_syn_ group; ^*&*^*P < 0.05*, ^*&&*^*P < 0.01*, compared with Yin_syn_ group

As shown in Fig. 1, compared with the CON group, the levels of weight, anal temperature, water intake, urine volume, and fecal water content in the BGZ group had no obvious changes, but the levels of anal temperature, urine volume, and cAMP were all significantly decreased (all *P < 0.05*) and accompanied with the significantly increased fecal water content (*P < 0.01*) in the Yang_syn_ group, while the levels of anal temperature, water intake, urine volume, and cAMP were all significantly increased (all *P < 0.05*) and accompanied with the significantly decreased fecal water content (*P < 0.05*) in the Yin_syn_ group. The change trends of behavioral characteristics and cAMP in the above-mentioned Yang_syn_/Yin_syn_ model rats were similar to the definition of Yang_syn_/Yin_syn_ in the diagnostic criteria of TCM syndromes. Under the intervention of BGZ, the levels of anal temperature, urine volume, and cAMP were all significantly increased (all *P < 0.05*) and accompanied by the significantly decreased fecal water content (*P < 0.05*) in the Yang_syn_+BGZ group compared with the Yang_syn_ group, while the levels of weight, water intake, urine volume, and cAMP were all significantly decreased (all *P < 0.05*) and accompanied with the significantly increased anal temperature and fecal water content (both *P < 0.05*) in the Yin_syn_+BGZ group compared with the Yin_syn_ group.

### Effect of BGZ treatment on the changes in organ indexes in Yin _syn_ and Yang _syn_ model rats

**Fig. 2:**
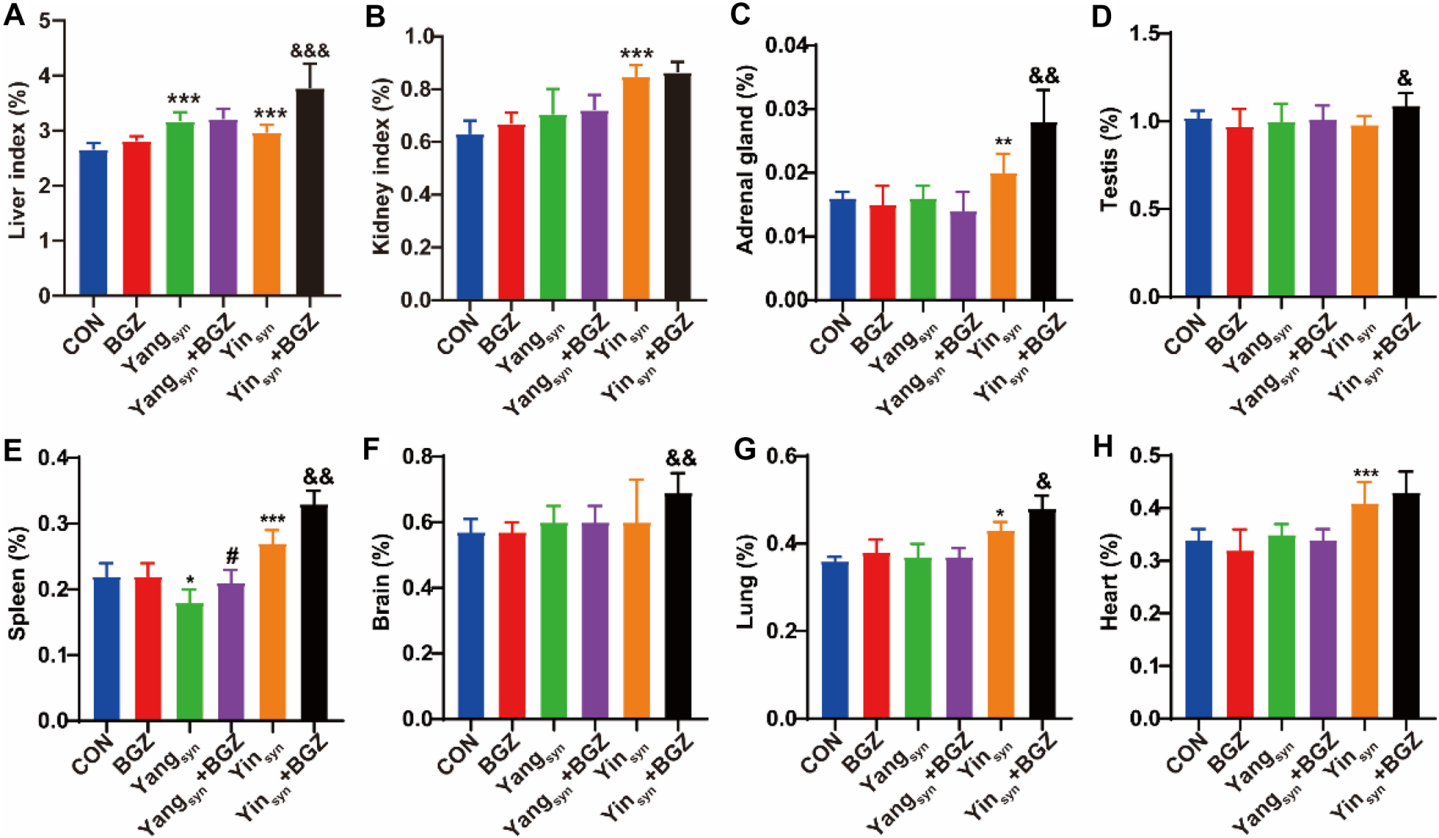
Effect of BGZ treatment on changes in organ indexes (N = 8). Liver (**A**), Kidney (**B**), Adrenal gland (**C**), Testis (**D**), Spleen (**E**), Brain (**F**), Lung (**G**) and Heart (**H**). *P < 0.05, **P < 0.01, ***P < 0.001, compared with CON group; ^*#*^*P < 0.05*, compared with Yang_syn_ group; ^*&*^*P < 0.05*, ^*&&*^*P < 0.01*, ^*&&&*^*P < 0.001*, compared with Yin_syn_ group

As shown in Fig. 2, compared with the CON group, all organ indexes in the BGZ group had no obvious changes, while the liver index was significantly increased (*P < 0.001*) and accompanied by the significantly increased spleen index (*P < 0.05*) in the Yang_syn_ group, but the organ indexes of the liver, kidney, adrenal gland, spleen, lung, and heart in the Yin_syn_ group were all significantly increased (all *P < 0.05*). Under the intervention of BGZ, the spleen index in the Yang_syn_+BGZ group was significantly increased (*P < 0.05*) compared with the Yang_syn_ group, while all organ indexes (except for kidney and heart) in the Yin_syn_+BGZ group were significantly increased compared with the Yin_syn_ group (all *P < 0.05*).

### Effect of BGZ treatment on the serum levels of ALT, AST, Scr, and liver histopathological changes in Yin _syn_ and Yang _syn_ model rats

**Fig. 3:**
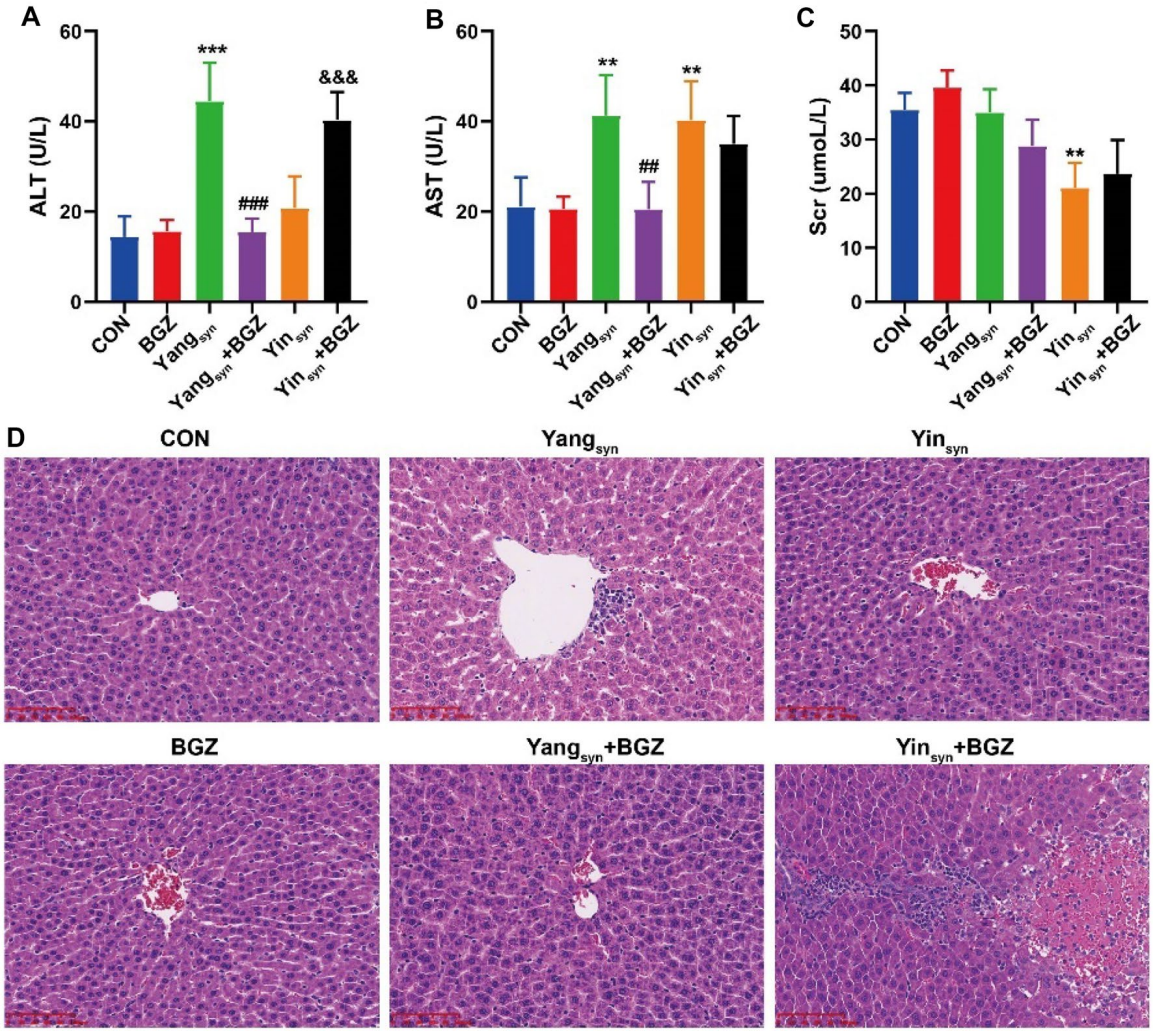
Effect of BGZ treatment on serum biochemistry and HE staining (N = 6 ~ 8). Serum ALT (**A**), AST (**B**), and Scr (**C**) activity. **P < 0.01, ***P < 0.001, compared with CON group; ^*##*^*P < 0.01*, ^*###*^*P < 0.001*, compared with Yang_syn_ group; ^*&&&*^*P < 0.001*, compared with Yin_syn_ group. (**D**) Typical histopathological section photographs of rat liver specimens for HE analysis (Scale bar, 100 μm)

Serum ALT, AST, and Scr, which are well-recognized markers of various types of liver and kidney damage, were used for the analysis. As for liver function (Fig. 3A–B), there were no obvious changes in the levels of ALT and AST between the BGZ group and the CON group. In contrast, the levels of ALT and AST in the Yang_syn_ group increased prominently, while BGZ significantly reversed the phenomena (all *P < 0.05*). Though the AST level in the Yin_syn_ group was significantly increased (*P < 0.01*), there was no obvious change in the levels of AST between the Yin_syn_+BGZ group and the Yin_syn_ group, while the ALT level was significantly increased after administration of BGZ in the Yin_syn_+BGZ group (*P < 0.001*), illustrating that there was a certain risk of liver injury in rats with Yin_syn_ after administration of BGZ. As for kidney function (Fig. 3C), no significant Scr changes were found in almost all groups except for the Yin_syn_ group (*P < 0.001*). Combined with the changes in the kidney organ index, it revealed that BGZ may have no obvious renal toxicity in rats with Yang_syn_ or Yin_syn_. As shown in Fig. 3D, the liver sections of the CON group showed normal hepatocyte structures. The liver samples from BGZ-treated rats were almost indistinguishable from normal rats. The liver samples from the Yang_syn_ group exhibited hepatocyte focal necrosis, loss of central vein intima, and inflammatory cell infiltration in portal vein areas, while the above symptoms were alleviated in the Yang_syn_+BGZ group. The liver samples from the Yin_syn_ group exhibited slight inflammatory infiltration in the portal area but no evident hepatocyte injury, while the hepatocyte focal necrosis and inflammatory cell infiltration were aggravated in the Yin_syn_+BGZ group. Combined with the levels of ALT, AST, liver index, and liver pathological examination, it was suggested that BGZ has a preferable liver protection effect on rats with Yang_syn_ and a certain risk of liver injury effect on rats with Yin_syn_.

### DEGs alterations of BGZ treatment in Yin_syn_ and Yang_syn_ model rats

**Fig. 4:**
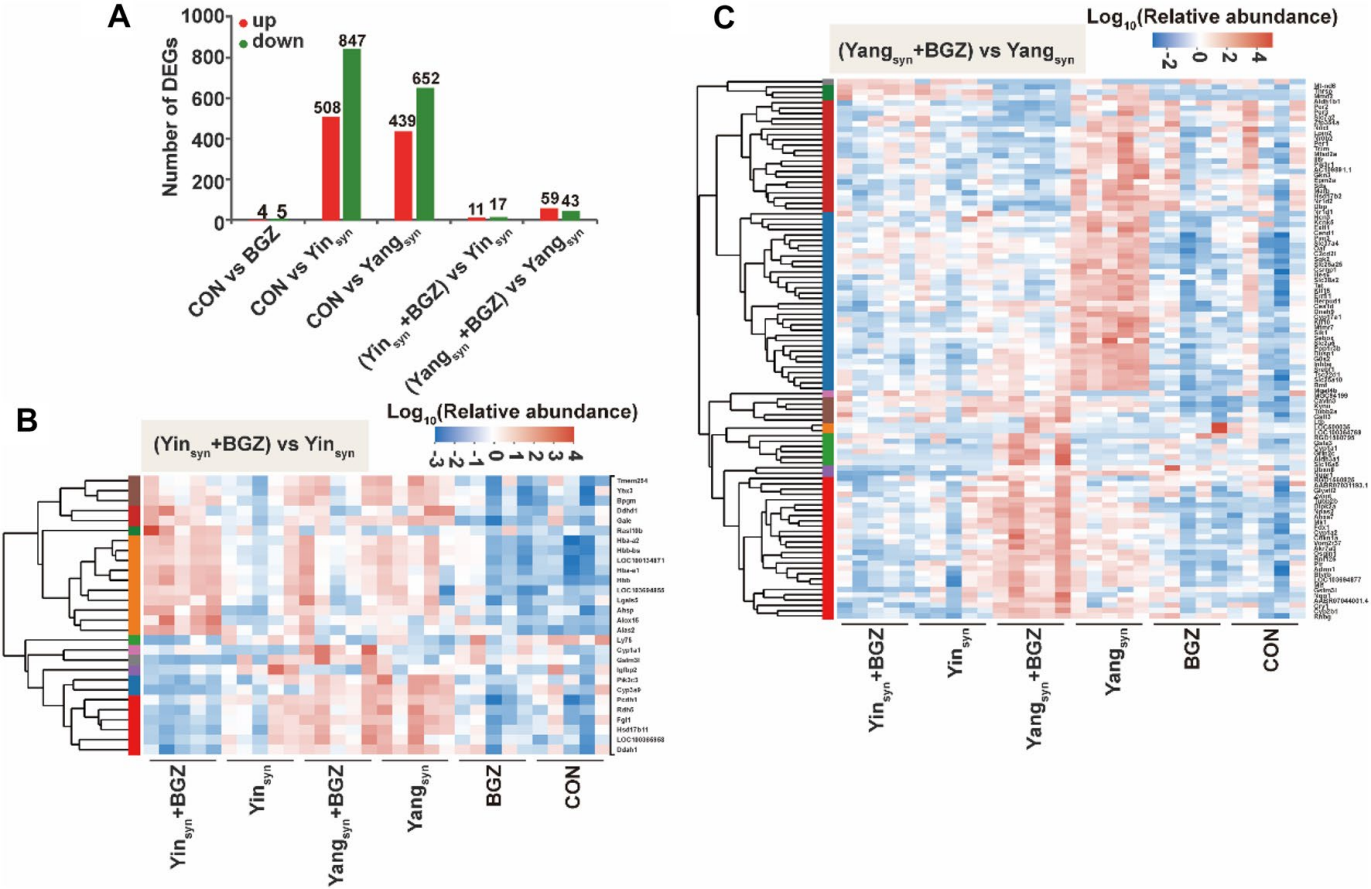
Transcriptomic alterations of BGZ treatment on Yin_syn_ and Yang_syn_ rats (N = 5). **A** The number of DEGs compared with each group. The red color represents up-regulated genes and the green color represents down-regulated genes. **B** Heatmap showing the intersection of DEGs between the Yin_syn_+BGZ group and the Yin_syn_ group. **C** Heatmap showing the intersection of DEGs between the Yang_syn_+BGZ group and the Yang_syn_ group

To reveal the mechanism of different therapeutic effects of BGZ on Yin_syn_ and Yang_syn_ rats, liver gene expression profiles were obtained from the CON, BGZ, Yang_syn_, Yang_syn_+BGZ, Yin_syn_, and Yin_syn_+BGZ groups using RNA-Seq analysis. As shown in Fig. 4A, compared with the CON group, BGZ barely influenced the gene expression with only 4 DEGs increased and 5 DEGs decreased, while 439 up-regulated DEGs and 652 down-regulated DEGs were found in the Yang_syn_ group, and 508 up-regulated DEGs along with 847 down-regulated DEGs were found in the Yin_syn_ group. Compared to the Yang_syn_ group, the number of up-regulated and down-regulated DEGs decreased to 59 and 43 by BGZ treatment, respectively (Additional file 1: Table S2), while for the Yin_syn_ group, the number of up-regulated and down-regulated DEGs decreased to 11 and 17 by BGZ treatment, respectively (Additional file 1: Table S3). To further display the above differences in DEGs more intuitively, heatmaps were constructed based on relative abundance (Fig. 4B, C).

### Correlation Analysis of the relative abundance of DEGs and the levels of serum biochemistry (ALT and AST)

**Fig. 5:**
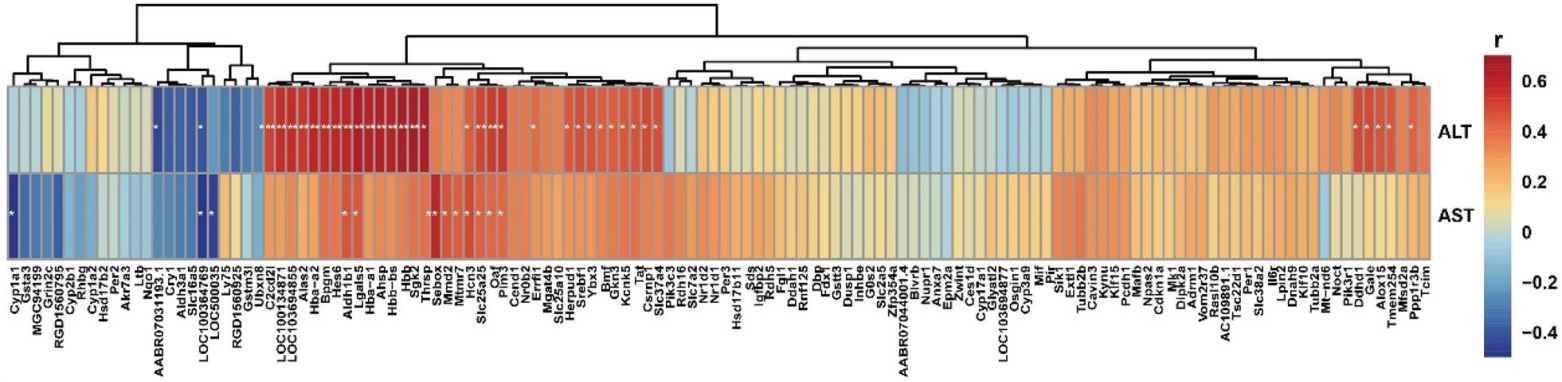
The relationship between the relative abundance of DEGs and the levels of serum biochemistry (ALT and AST) (N = 5). Color depth represents correlation strength, the red color represents positive correlation, and the blue color represents negative correlation. *P < 0.05, **P < 0.01, ***P < 0.001

By analyzing the correlations between serum biochemistry (ALT and AST) and DEGs changed using BGZ in Yin_syn_ and Yang_syn_ rats (Fig. 5), the results demonstrated that 36 DEGs, including 34 positive DEGs and 2 negative DEGs, were significantly correlated with ALT, and 12 DEGs, including 9 positive DEGs and 3 negative DEGs, were significantly correlated with AST. Among them, hyperpolarization-activated cyclic nucleotide-gated potassium channel 3 (Hcn3), aldehyde dehydrogenase 1 family member B1 (Aldh1b1), galectin 5 (Lgals5), solute carrier family 25 member 25 (Slc25a25), Pim-3 proto-oncogene, serine/threonine kinase (Pim3), and out at first (Oaf) were all significantly positively correlated with ALT and AST, and LOC100364769 was significantly negatively correlated with ALT and AST. The detailed relationship between DEGs and serum biochemistry (ALT and AST) was shown in Additional file 1: Table S4.

### *ROC curve analysis of DEGs and RT-qPCR verification*

**Fig. 6:**
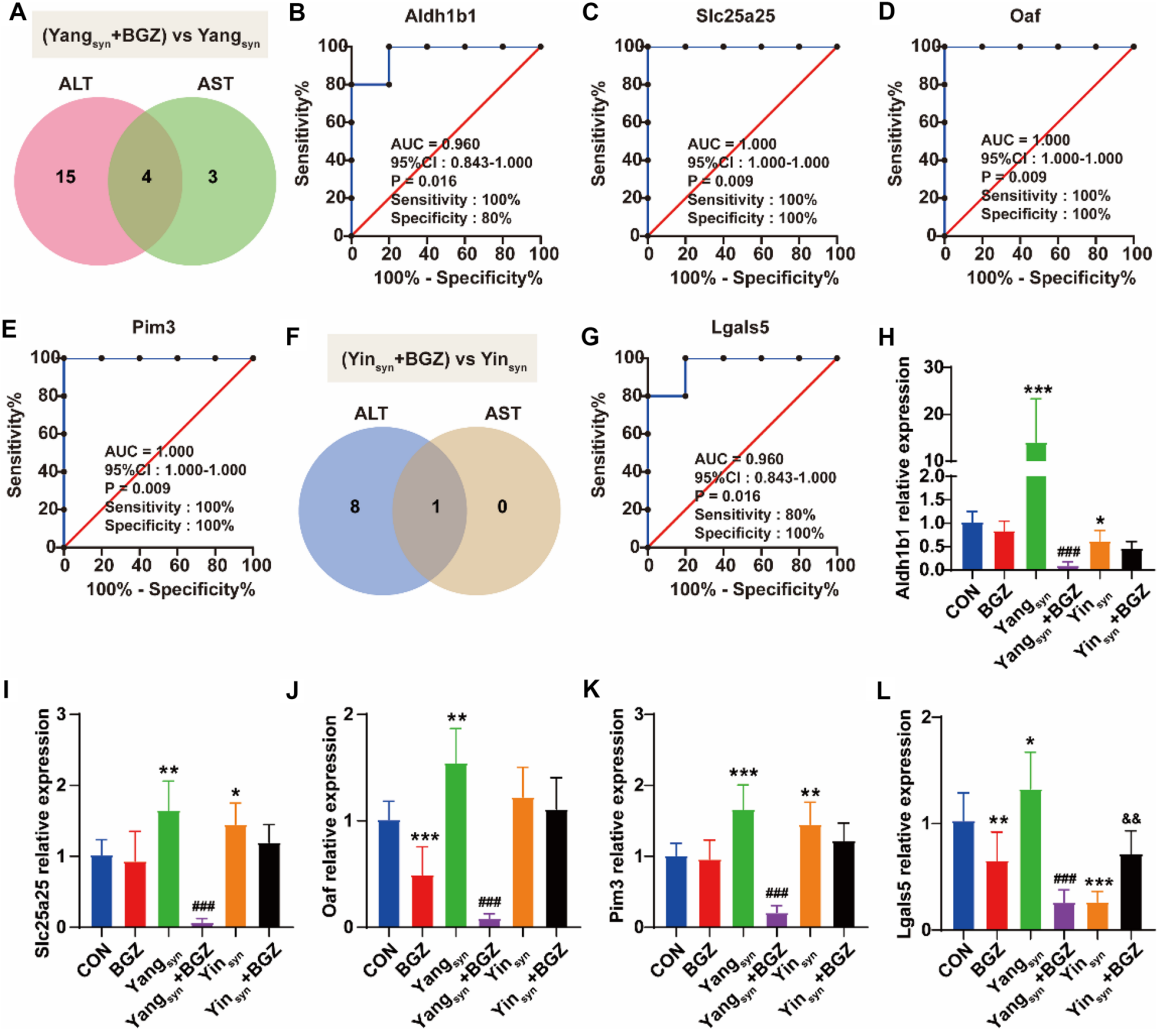
ROC analysis of DEGs associated with ALT and AST expression and RT-qPCR verification. **A** Venn diagram showing the number of DEGs associated with the levels of ALT and AST under ROC ≥ 0.90 between the Yang_syn_+BGZ group and the Yang_syn_ group. **B**–**E** ROC curves of DEGs associated with the levels of ALT and AST under ROC ≥ 0.90 between the Yang_syn_+BGZ group and Yang_syn_ group, Aldh1b1 (**B**), Slc25a25 (**C**), Oaf (**D**), and Pim3 (**E**). **F** Venn diagram showing the number of DEGs associated with the levels of ALT and AST under ROC ≥ 0.90 between the Yin_syn_+BGZ group and the Yin_syn_ group. **G** ROC curves of Lgals5 between the Yin_syn_+BGZ group and the Yin_syn_ group. **H**–**L** The mRNA expression of Aldh1b1 (**H**), Slc25a25 (**I**), Oaf (**J**), Pim3 (**K**), and Lgals5 (**L**). *P < 0.05, **P < 0.01, ***P < 0.001, compared with CON group; ^*###*^*P < 0.001*, compared with Yang_syn_ group; ^*&&*^*P < 0.01*, compared with Yin_syn_ group

To further explore the diagnostic efficacy of DEGs, a ROC curve analysis was performed using GraphPad Prism software (version 8.01). The results revealed that 22 DEGs could be better discriminated between the Yang_syn_+BGZ group and Yang_syn_ group, and 10 DEGs had better discrimination between the Yin_syn_+BGZ group and Yin_syn_ group (all the areas under the curve (AUC) of the ROC curves ≥ 0.9 and *P < 0.05*) (Additional file 1: Fig. S1). Venn analysis showed the ROC results of DEGs and their correlation with the levels of ALT and AST between the Yang_syn_+BGZ group and Yang_syn_ group (Fig. 6A), revealing that Aldh1b1, Slc25a25, Pim3, and Oaf can be used as potential biomarkers for the treatment of Yang_syn_ (Fig. 6B–E). Venn analysis also showed the ROC results of DEGs and their correlation with ALT and AST between the Yin_syn_+BGZ group and Yin_syn_ group (Fig. 6F), revealing that Lgals5 may be used as a potential biomarker for the treatment of Yin_syn_ (Fig. 6G). RT-qPCR verification of the above marker genes revealed that, as shown in Fig. 6H–L, compared with the CON group, the expression of Aldh1b1, Slc25a25, Pim3, Oaf, and Lgals5 in rats with Yang_syn_ were all significantly increased (all *P < 0.05*), while the levels of Aldh1b1 and Lgals5 were both decreased (both *P < 0.05*) and accompanied with the significantly increased Slc25a25 and Pim3 (both *P < 0.05*) in the Yin_syn_ group. Under BGZ intervention, the levels of Aldh1b1, Slc25a25, Pim3, Oaf, and Lgals5 were all significantly decreased in the Yang_syn_+BGZ group compared with the Yang_syn_ group (all *P < 0.001*), while only Lgals5 was significantly increased in rats with Yin_syn_+BGZ compared with the Yin_syn_ group (*P < 0.05*).

### GO and KEGG functional enrichment analysis of DEGs

**Fig. 7:**
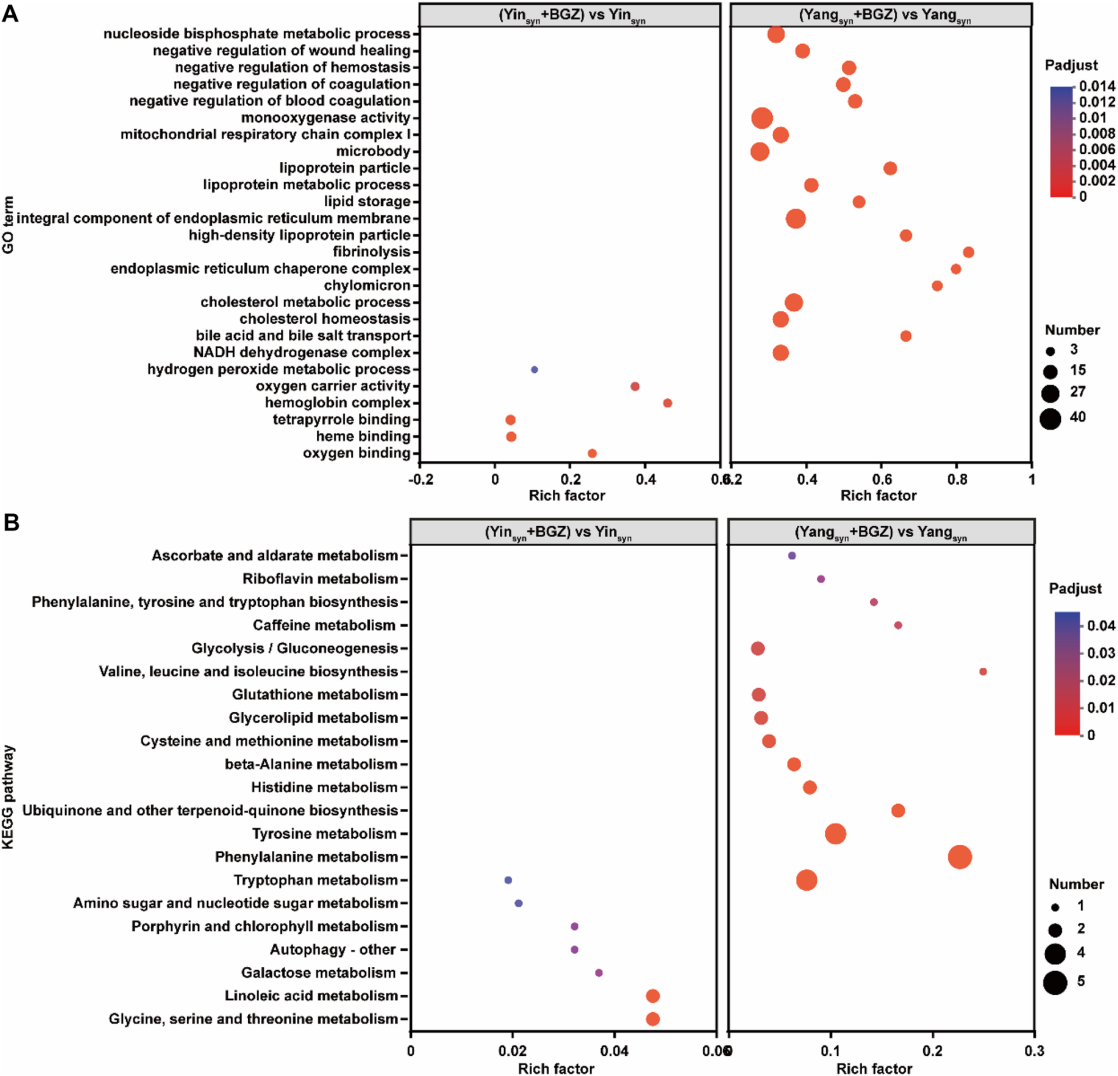
GO and KEGG functional enrichment pathways analysis of DEGs. **A** GO enrichment analysis. **B** KEGG enrichment analysis

GO functional enrichment analysis found that 95 GOs (Additional file 1: Table S5) were significantly changed in the Yang_syn_+BGZ group compared with the Yang_syn_ group, such as GO:0009991 (response to extracellular stimulus), GO:0032922 (circadian regulation of gene expression), GO:0031667 (response to nutrient levels), and GO:0009892 (negative regulation of metabolic process), while 6 GOs including GO:0019825 (oxygen binding), GO:0020037 (heme-binding), GO:0046906 (tetrapyrrole binding), GO:0005833 (hemoglobin complex), GO:0005344 (oxygen carrier activity), and GO:0042743 (hydrogen peroxide metabolic process) were significantly changed in the Yin_syn_+BGZ group compared with the Yin_syn_ group. However, there is no intersection in the GO terms of regulating liver function in rats with Yang_syn_/Yin_syn_ by BGZ, Fig. 7A only displays the top 20 GO terms of the DEGs enrichment levels based on the value of *Padjust < 0.05*, respectively.

KEGG functional enrichment analysis found it mainly involves 15 pathways, including amino acid metabolism (phenylalanine metabolism, tyrosine metabolism, tryptophan metabolism, histidine metabolism, cysteine and methionine metabolism, valine, leucine and isoleucine biosynthesis, phenylalanine, tyrosine and tryptophan biosynthesis), carbohydrate metabolism (glycolysis/gluconeogenesis, ascorbate and aldarate metabolism), lipid metabolism (glycerolipid metabolism), were significantly changed in the Yang_syn_+BGZ group compared with the Yang_syn_ group. while 7 pathways, including amino acid metabolism (tryptophan metabolism), carbohydrate metabolism (galactose metabolism), lipid metabolism (arachidonic acid metabolism), metabolism of cofactors and vitamins (porphyrin and chlorophyll metabolism), were significantly changed in the Yin_syn_+BGZ group compared with the Yin_syn_ group. However, the KEGG pathway of BGZ regulating liver function in rats with Yang_syn_/Yin_syn_ has only one intersection (tryptophan metabolism). Figure 7B only shows the top 20 KEGG pathways of the DEGs enrichment degree according to the value of *Padjust < 0.05*, respectively.

### Metabolomic analysis of BGZ treatment in Yin_syn_ and Yang_syn_ model rats

**Fig. 8:**
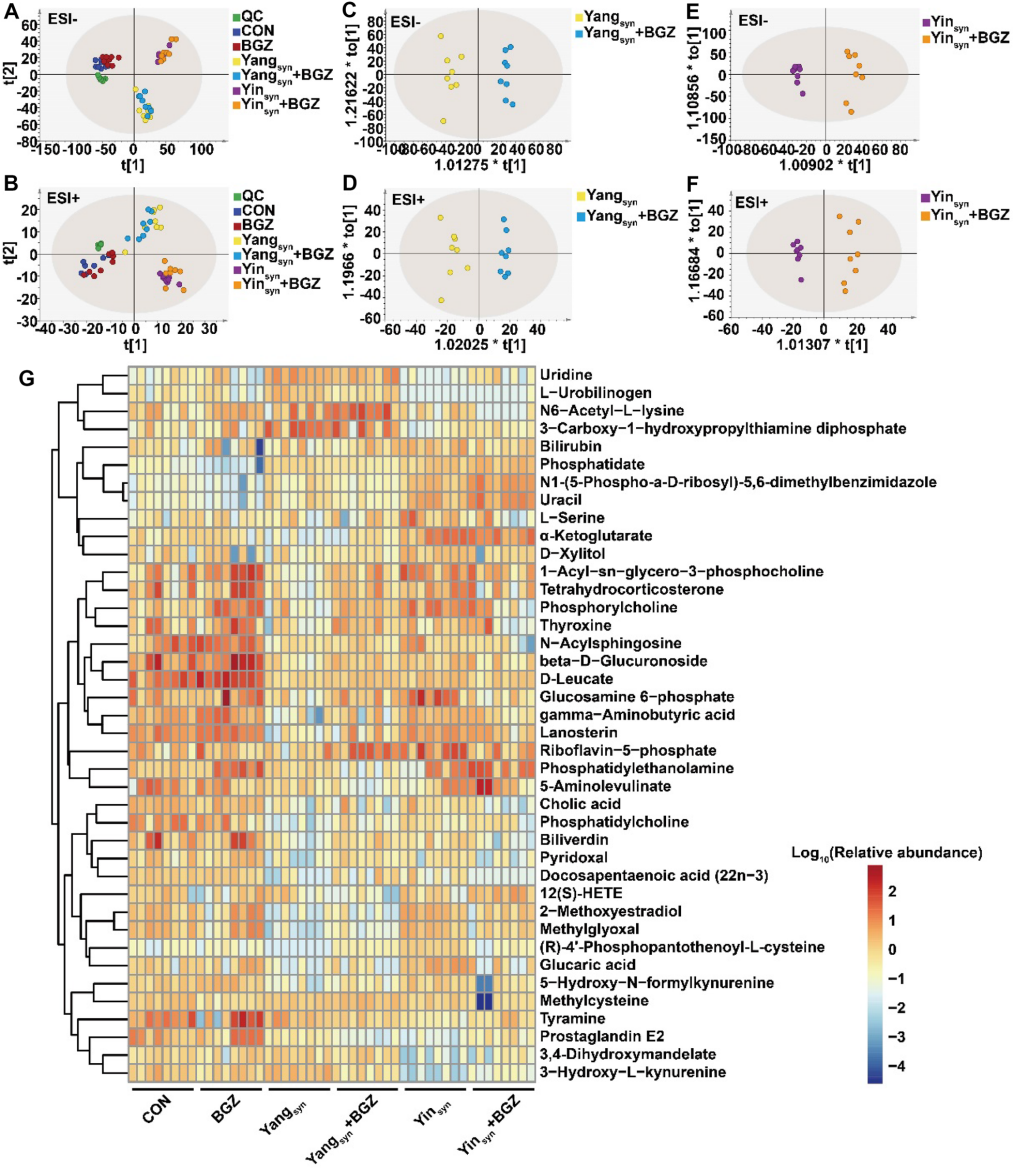
Metabolomic alterations of BGZ treatment on Yin_syn_ and Yang_syn_ rats (N = 8). **A**–**B** Scatter plot of liver metabolites in all groups determined by PCA in ESI- mode (**A**) and ESI + mode (**B**). **C**–**D** Scatter plot of liver metabolites between the Yang_syn_+BGZ and Yang_syn_ groups determined by OPLS-DA in ESI- mode (**C**) and ESI + mode (**D**). **E**–**F** Scatter plot of liver metabolites between the Yin_syn_+BGZ and Yin_syn_ groups determined by OPLS-DA in ESI- mode (**E**) and ESI + mode (**F**). **G** Heatmap of the metabolites identified. Color depth represents the variation trend of the relative abundance of metabolites, the red color represents up-regulated metabolites, and the blue color represents down-regulated metabolites

As shown in Fig. 8A and B, the QC samples gathered closely in both PCA score plots, indicating the stability of the UPLC-QTOF/MS system throughout the analysis. The CON, Yang_syn_, and Yin_syn_ groups can be well distinguished, suggesting that the metabolic information between the Yang_syn_ and Yin_syn_ groups has been changed. OPLS-DA analysis found that the comparison between the Yang_syn_ group and the Yang_syn_+BGZ groups (Fig. 8C and D) could both be significantly separated under ESI + and ESI- modes, and the comparison between the Yin_syn_ group and Yin_syn_+BGZ group (Fig. 8E, F) could also be significantly separated under ESI + and ESI- modes, and the permutation tests for OPLS-DA analysis revealed the models were not overfitted (Additional file 1: Fig. S2), indicating that significant metabolic disturbance occurred in the Yang_syn_+BGZ group and Yin_syn_+BGZ group after BGZ treatment. A total of 40 metabolites were identified based on *m/z* and corresponding secondary fragment ion characteristic maps (Table 1, Additional file 1: Fig. S3). Among the metabolites, 27 metabolites were markedly changed in the Yang_syn_+GBZ group compared with the Yang_syn_ group, and 31 metabolites were significantly changed in the Yin_syn_+GBZ group compared with the Yin_syn_ group. The heatmap for the above differential metabolites is presented in Fig. 8G.

**Table 1:** Identified differential metabolites

ESI	m/z	RT (min)	KEGG	Metabolite	(Yang_syn_+BGZ) vs. (Yang_syn_)	(Yin_syn_+BGZ) vs. (Yin_syn_)
−	145.0132	0.9378	C00026	α-Ketoglutarate	↑*	↓
−	455.0965	2.9226	C00061	Riboflavin-5-phosphate	↑*	↓*
−	104.0348	0.8028	C00065	L-Serine	↑	↓*
+	113.0352	0.9136	C00106	Uracil	↓*	↑*
+	778.5407	13.2262	C00157	Phosphatidylcholine	↑	↓
−	582.5100	13.2602	C00195	N-Acylsphingosine	↑**	↓*
−	148.0378	1.3859	C00250	Pyridoxal	↑*	↓*
−	243.0615	1.3509	C00299	Uridine	↓	↑*
−	102.0556	0.8313	C00334	gamma-Aminobutyric acid	↑*	↓*
−	766.6037	9.3765	C00350	Phosphatidylethanolamine	↓**	↑*
−	240.0224	1.5073	C00352	Glucosamine 6-phosphate	↑*	↓**
−	151.0569	0.9235	C00379	D-Xylitol	↑	↓*
−	671.4653	13.3460	C00416	Phosphatidate	↓***	↑*
+	173.0922	0.8994	C00430	5-Aminolevulinate	↓**	↑
+	160.0756	1.9497	C00483	Tyramine	↓*	↑*
−	583.2469	8.6720	C00486	Bilirubin	↑	↓*
+	583.2554	8.0917	C00500	Biliverdin	↑**	↓*
+	90.0555	0.8565	C00546	Methylglyoxal	↑	↓*
−	397.2291	11.6884	C00584	Prostaglandin E2	↓**	↑*
−	229.0667	1.6209	C00588	Phosphorylcholine	↑*	↓*
+	426.3162	15.2409	C00695	Cholic acid	↑	↓**
+	252.0724	2.1330	C00818	Glucaric acid	↑	↓*
+	465.3556	11.5639	C01724	Lanosterin	↑	↓*
+	799.6627	15.2528	C01829	Thyroxine	↑**	↓
+	196.0943	4.0050	C03264	D-Leucate	↑**	↓
−	187.1076	1.3080	C02727	N6-Acetyl-L-lysine	↑*	↓*
+	560.3333	8.4013	C03033	beta-D-Glucuronoside	↑*	↓
−	205.0645	1.7274	C03227	3-Hydroxy-L-kynurenine	↓*	↑*
+	522.3564	9.5634	C04230	1-Acyl-sn-glycero-3-phosphocholine	↑**	↓
−	401.0785	6.4160	C04352	(R)-4’-Phosphopantothenoyl-L-cysteine	↑**	↓***
+	381.0794	0.9636	C04778	N1-(5-Phospho-a-D-ribosyl)-5,6-dimethylbenzimidazole	↓*	↑**
−	347.1835	8.9063	C05302	2-Methoxyestradiol	↑	↓**
−	526.0585	1.3859	C05381	3-Carboxy-1-hydroxypropylthiamine diphosphate	↓	↑*
+	389.2142	4.7813	C05476	Tetrahydrocorticosterone	↑**	↓
−	183.0346	0.8099	C05580	3,4-Dihydroxymandelate	↓	↑*
−	297.0703	0.9593	C05648	5-Hydroxy-N-formylkynurenine	↑	↓*
−	577.3444	13.7089	C05789	L-Urobilinogen	↓*	↑
−	365.2324	6.4089	C14777	12(S)-HETE	↓*	↑*
+	353.2472	6.4221	C16513	Docosapentaenoic acid (22n-3)	↑*	↓*
+	136.0396	1.6256	C22040	Methylcysteine	↑*	↓**

*P < 0.05, **P < 0.01, ***P < 0.001

### Correlation analysis of the relative abundance of metabolites and the levels of serum biochemistry (ALT and AST)

**Fig. 9:**
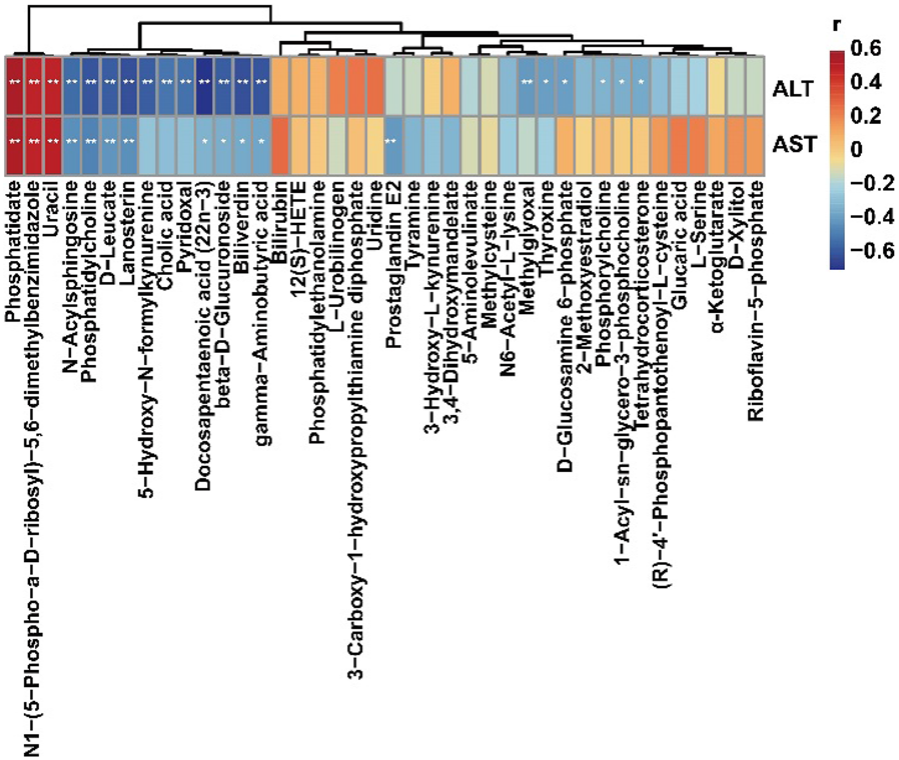
Heatmap of correlation between the levels of serum biochemistry (ALT and AST) and the relative abundance of metabolites (N = 6 ~ 8). The color depth represents correlation strength, the red color represents positive correlation, and the blue color represents negative correlation. *P < 0.05, **P < 0.01, ***P < 0.001

By analyzing the correlations between serum biochemistry (ALT and AST) and differential metabolites (Figs. 9), 20 metabolites, including 3 positive and 17 negative correlations, were significantly correlated with ALT, and 12 metabolites, including 3 positive and 9 negative correlations, were significantly correlated with AST. The correlation coefficient r and *P-value* are shown in Additional file 1: Table S6. Among them, Phosphatidate, Uracil, Prostaglandin E2, and N1-(5-Phospho-a-D-ribosyl)-5,6-dimethylbenzimidazole were all significantly positively correlated with ALT and AST, and Phosphatidylcholine, Lanosterin, N-Acylsphingosine, D-Leucate, Gamma-aminobutyric acid, Biliverdin, beta-D-Glucuronoside, and Docosapentaenoic acid (22n-3) were all significantly negatively correlated with ALT and AST.

### ROC curve analysis of metabolites

**Fig. 10:**
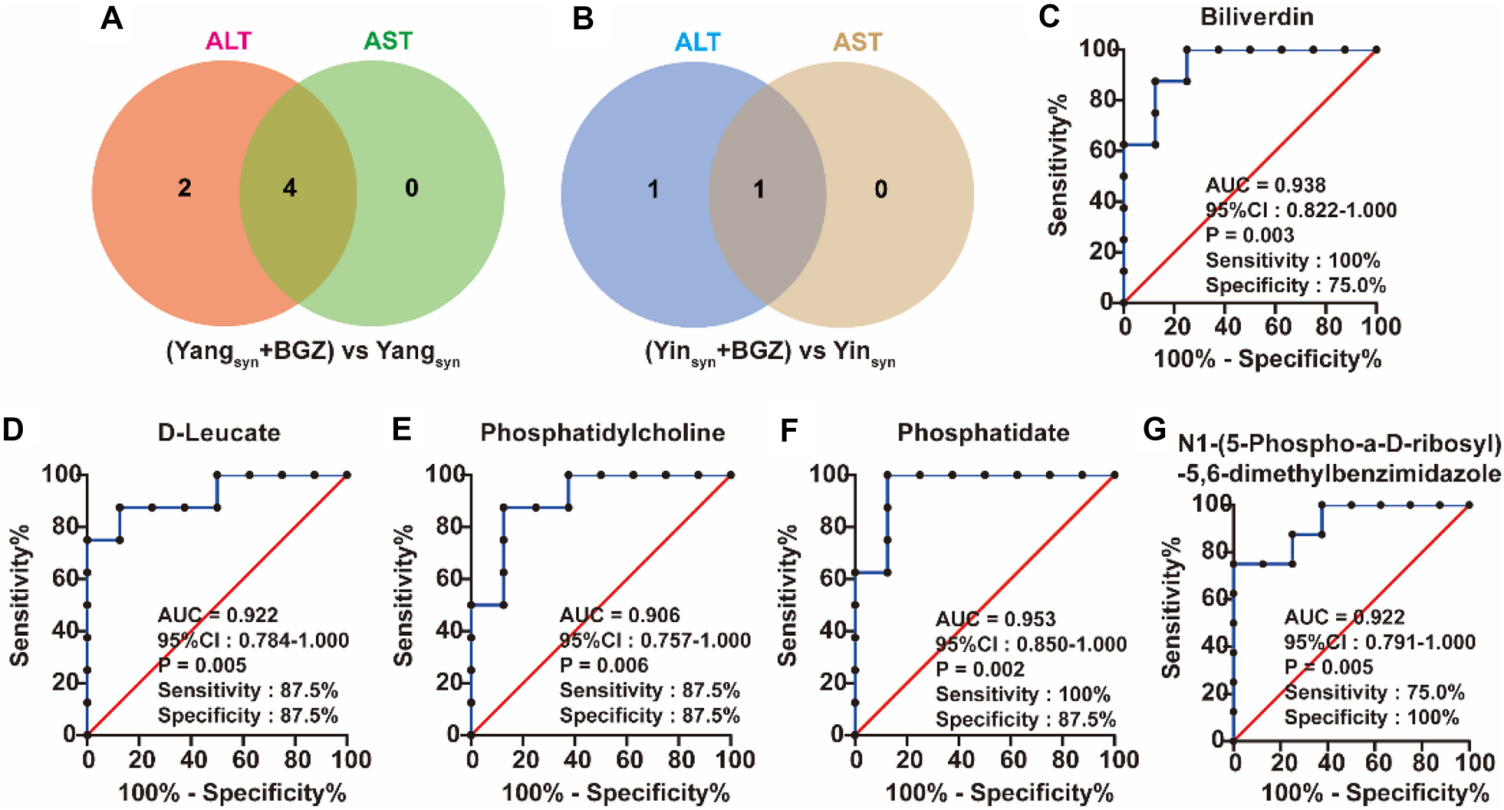
ROC analysis of metabolites associated with ALT and AST expression (N = 8). **A**–**B** Venn diagram showing the number of metabolites associated with the levels of ALT and AST under ROC ≥ 0.90 among the comparison of the Yang_syn_+BGZ group and Yang_syn_ group (**A**) and the comparison of the Yin_syn_+BGZ group and the Yin_syn_ group (**B**). **C**–**G** ROC curves of the above metabolites (Biliverdin (**C**), D-Leucate (**D**), Phosphatidylcholine (**E**), Phosphatidate (**F**), and ROC curves of N1-(5-Phospho-a-D-ribosyl)-5,6-dimethylbenzimidazole (G)) associated with the levels of ALT and AST under ROC ≥ 0.90.

As shown in Additional file 1: Figs. S4, 9 metabolites had better discrimination between the Yang_syn_+BGZ group and Yang_syn_ group, and 5 metabolites had better discrimination between the Yin_syn_+BGZ group and Yin_syn_ group (all the areas under the curve (AUC) of the ROC curves ≥ 0.9 and *P < 0.05*). Venn analysis showed the ROC results of metabolites and their correlation with the levels of ALT and AST among the comparison of the Yang_syn_+BGZ group and Yang_syn_ group (Fig. 10A) and the comparison of the Yin_syn_+BGZ group and the Yin_syn_ group (Fig. 10B). Among them, Phosphatidate, Phosphatidylcholine, D-Leucate, and Biliverdin may be used as potential hepatoprotective biomarkers for the treatment of Yang_syn_ (Fig. 10C–E), while N1-(5-Phospho-a-D-ribosyl)-5,6-dimethylbenzimidazole may be used as a potential hepatotoxicity biomarker for the treatment of Yin_syn_ (Fig. 10G).

### Comparison of overall metabolic profiles

**Fig. 11:**
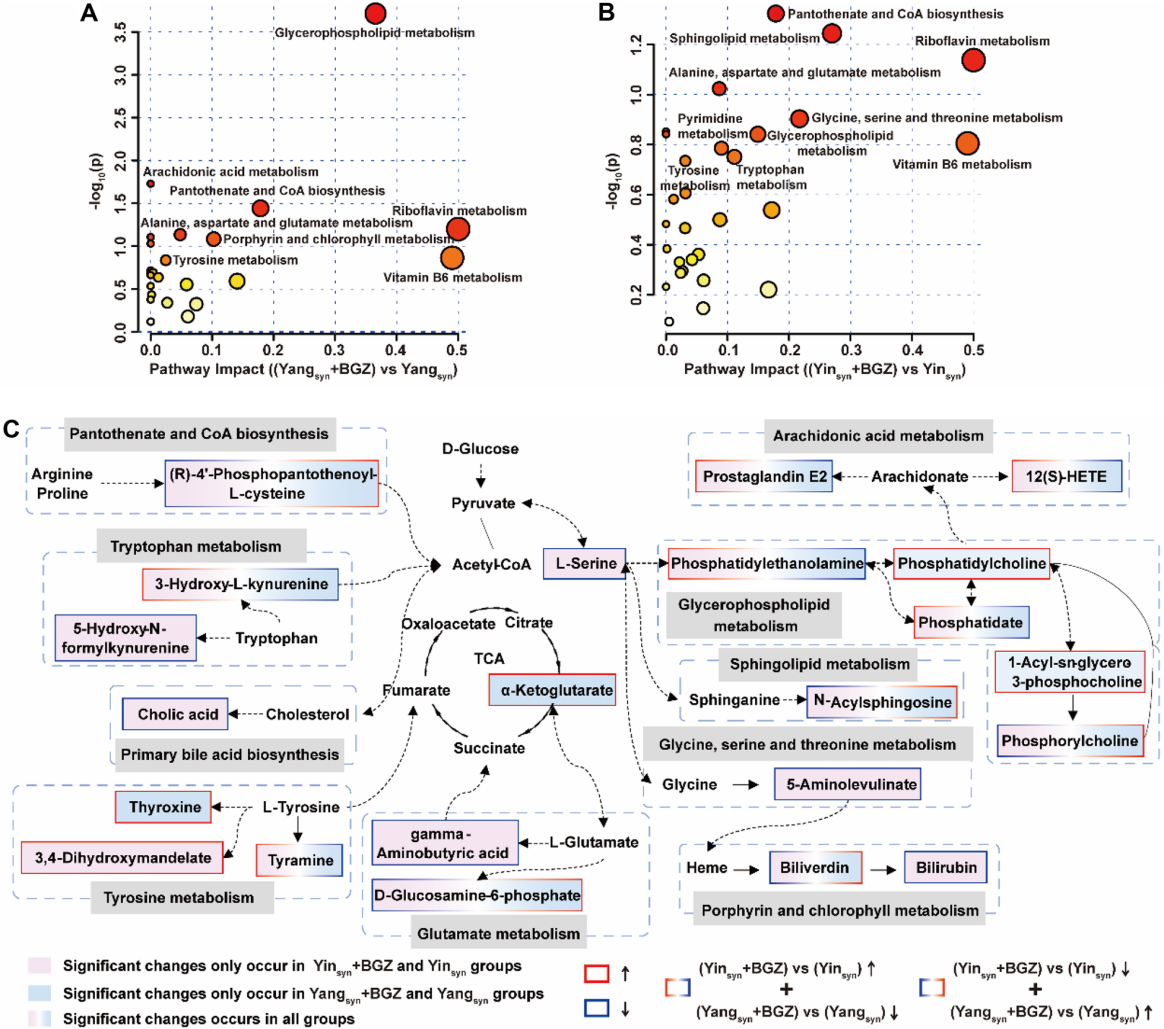
Overall metabolic profile. **A**–**B** Schematic diagram of the disturbed metabolic pathways for the BGZ treatment on Yang_syn_ (**A**) and Yin_syn_ (**B**) rats. **C** Correlation network diagram of DEGs and metabolism of BGZ intervention in Yin_syn_ and Yang_syn_ rats

To further understand the metabolic disorder of susceptible syndromes, the KEGG identification code of differential metabolites was used for pathway analysis via MetaboAnalyst 5.0 (https://www.metaboanalyst.ca/). The schematic diagram of the disturbed metabolic pathways changed by the treatment of BGZ for Yang_syn_ and Yin_syn_ rats is presented in Fig. 11A and B. The redder the bubble color, the smaller the *P* value of the metabolic pathway. The details for the value of *P* are shown in Additional file 1: Tables S7 and S8. The results showed that the metabolites of BGZ regulating liver function in rats with Yang_syn_ mainly involved glycerophospholipid metabolism, arachidonic acid metabolism, pantothenate and coenzyme A (CoA) biosynthesis. The metabolites of BGZ regulating liver function in rats with Yin_syn_ mainly involve pantothenate and the CoA biosynthesis, glycerophospholipid metabolism. To compare the differences in metabolic profiles for BGZ treatment on Yin_syn_ and Yang_syn_ rats, a network map was constructed based on the identified metabolites and enriched metabolic pathways. As shown in Fig. 11C, metabolic pathways and metabolites interacted with each other to form a complex network.

### Integration analysis of “metabolites-genes-pathways” regulated by BGZ in rats with Yin_syn_ and Yang_syn_

**Fig. 12:**
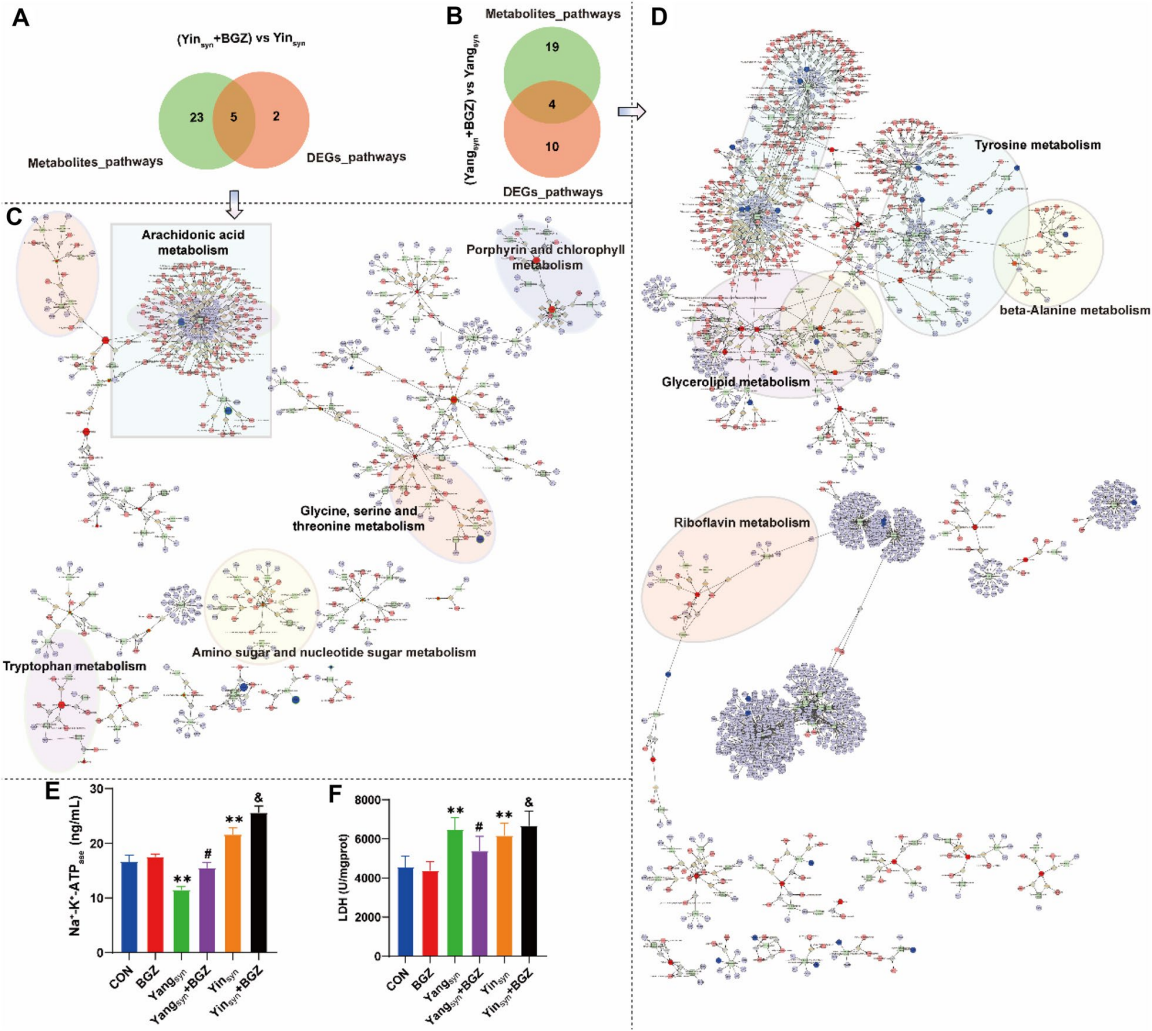
Integration analysis of “metabolites-genes-pathways” regulated by BGZ in rats with Yin_syn_ and Yang_syn_. **A**–**B** Venn analysis of the crosstalk pathways of co-regulation of metabolites and DEGs by BGZ in Yin_syn_ (**A**) and Yang_syn_ (**B**) rats, **C**–**D** Visualization of the network relationship among the “metabolite-gene-pathway” regulated by BGZ in rats with Yin_syn_ (**C**)and Yang_syn_ (**D**). **E**–**F** Effect of BGZ treatment on the changes in the expression of Na^+^-K^+^-ATP_ase_ (**E**) and LDH (**F**) (N = 6 ~ 8). *P < 0.05, **P < 0.01, compared with CON group; ^*#*^*P < 0.05*, compared with Yang_syn_ group; ^*&*^*P < 0.05*, compared with Yin_syn_ group

In the Venn analysis of the metabolite enrichment pathways and DEGs enrichment pathways, it was found that the pathways of BGZ regulating liver function in rats with Yin_syn_+BGZ were mainly involved in the glycine, serine and threonine metabolism, porphyrin and chlorophyll metabolism, arachidonic acid metabolism, amino sugar and nucleotide sugar metabolism, tryptophan metabolism (Fig. 12A), while the pathways regulated by BGZ in rats with Yang_syn_+BGZ were mainly involved in the glycerolipid metabolism, tyrosine metabolism, riboflavin metabolism, beta-alanine metabolism (Fig. 12B). Figure 12 C and 12D visualized the network relationship among the “metabolites-genes-pathways” regulated by BGZ in rats with Yin_syn_ and Yang_syn_, respectively. It is worth noting that, the pathways of BGZ regulating in rats with Yin_syn_ and Yang_syn_ are almost all involved in energy metabolism, amino acid metabolism, lipid metabolism, and metabolism of cofactors and vitamins, and the above pathways were also cross-involved in Na^+^-K^+^-ATP_ase_ and LDH [49, 50]. As shown in Fig. 12E F, the study further found the expression Na^+^-K^+^-ATP_ase_ was significantly decreased in the Yang_syn_ group (*P < 0.01*) but increased in the Yin_syn_ group (*P < 0.01*) while compared with the CON group. In addition, the levels of LDH were significantly increased in both model groups (both *P < 0.01*). Under BGZ intervention, the levels of Na^+^-K^+^-ATP_ase_ in the Yang_syn_+BGZ group and Yin_syn_+BGZ group were both significantly increased when compared with corresponding model rats, respectively (both *P < 0.05*), while the levels of LDH in the Yang_syn_+BGZ group and Yin_syn_+BGZ group showed the opposite trends of change when compared with corresponding model rats, respectively (both *P < 0.05*).

## Discussion

Individual variability in liver injury following drug ingestion is a major challenge for DILI research and clinical prevention [1]. In the study, it was found that Yin_syn_ and Yang_syn_ are two different predisposed individual states of hepatotoxicity or hepatoprotection caused by BGZ. From the perspective of the gene expression profile, BGZ mostly does not affect normal rats, but it has a mild or greater impact on the change numbers of DEGs in Yin_syn_ (28 DEGs) and Yang_syn_ (102 DEGs) rats, respectively. From the perspective of endogenous metabolites, Yin_syn_ and Yang_syn_ rats can be well distinguished on the PCA scatter plot, and the OPLS-DA plot can further better distinguish Yin_syn_, Yang_syn_, and their corresponding BGZ-treated groups, suggesting that BGZ exerts different metabolic perturbation patterns for different models.

Among the DEGs with ROC ≥ 0.9 and significant correlation with ALT and AST, Slc25a25 may function as an ATP-Mg/Pi carrier to mediate the transport of Mg-ATP in exchange for phosphate. It is also likely responsible for the net uptake or efflux of adenine nucleotides into or from the mitochondria and is highly expressive in acute liver failure or induced by exogenous thyroxine [51, 52]. This study confirmed the high expression of Slc25a25 in the liver of Yin_syn_ and Yang_syn_ rats, which is consistent with the phenomenon of abnormal liver function [52]. However, only the expression of Slc25a25 in Yang_syn_ rats was inhibited by BGZ, indicating that Slc25a25 may be a hepatoprotective gene for BGZ in Yang_syn_ rats, and the way to aggravate liver injury in rats with Yin_syn_ may not by regulating Slc25a25. Pim3 is a liver growth-stimulating factor with serine/threonine kinase activity and is involved in gp130-mediated induction of cell proliferation and protection of apoptosis downstream of signal transducer and activator of transcription 3 (STAT3) [53, 54]. Pim3 is barely expressed in normal tissues but is highly expressed in the prostate, large intestine, liver, and other cancer tissues [55]. Therefore, its expression level is mainly used to evaluate tumor expression and metastasis [56, 57]. Pim3 is a kind of aldosterone regulatory protein with the ability to promote the expression of aldosterone [58], and the abnormal increase of aldosterone can increase the blood pressure of patients [59], which can result in the occurrence of heart, liver, and kidney damage in severe cases [60]. This study also found that the expression of Pim3 was positively correlated with the expression of ALT, which also suggested that the high expression of Pim3 may be another potential target for liver injury. Surprisingly, BGZ only decreased the expression of Pim3 in Yang_syn_ rats with almost no change in Yin_syn_ rats. The above results indicated that Pim3 may be a marker gene for BGZ to exert a hepatoprotective effect on Yang_syn_ rats. In addition, BGZ may also have a potential therapeutic effect on tumor patients with Yang_syn_, and the anti-tumor effect of BGZ has been confirmed [61, 62].

Among the metabolites with ROC ≥ 0.90 and significant correlation with ALT and AST, glycerophospholipids (including phosphatidylcholine, phosphatidylethanolamine, and phosphatidylserine) are the most abundant phospholipids in the body. In addition to constituting biofilm, they are also one of the components of bile and membrane surface-active substances and participate in protein recognition and signal transduction through the cell membrane [63]. Hepatocytes can express various glycerophospholipids activities, making the liver an important organ for glycerophospholipid metabolism. Dysregulation of glycerophospholipids is related to the development and progression of liver diseases including hepatitis, liver cancer, fatty liver, and liver fibrosis [64]. For example, the abnormal change in the ratio of phosphatidylcholine to phosphatidylethanolamine will affect energy metabolism and is closely related to disease progression [65]. The changes in hepatic phospholipid composition are also associated with fatty liver disease and impaired postoperative liver regeneration [66–68].

In the glycerolipid metabolic pathway, phosphatidate can activate hepatic interleukin 6 (IL-6) signaling through inter-organ crosstalk and alleviate acetaminophen-induced liver injury in mice [68]. Phosphatidate also acts as an ionophore in the brain between depolarization and the release of neurotransmitters such as gamma-Aminobutyric Acid (GABA) [69]. The increase in the level of GABA can enhance the body’s immunity under stress conditions [70] and has a good protective effect on acute liver injury or liver failure induced by ethanol, fluoride, and d-galactosamine [71–73]. In the study, under the intervention of BGZ, the expression of PE and GABA in the liver of rats with Yin_syn_ and Yang_syn_ both showed opposite trends, suggesting that regulating glycerophospholipid metabolism may be another way in which BGZ exerts liver damage and protection in Yin_syn_ and Yang_syn_ rats, respectively.

In the arachidonic acid metabolic pathway, 12(S)-HETE, as an important inflammatory marker, can affect the inflammatory process by stimulating the release of cytokines such as tumor necrosis factor alpha-like (TNF-α) and IL-6. Blocking the production of 12(S)-HETE can inhibit ischemia-reperfusion-induced liver dysfunction, inflammation, and cell death [74]. As a regulatory enzyme of arachidonic acid metabolism, arachidonate 15-lipoxygenase (Alox15) can catalyze it to 12(S)-HPETE, which is further reduced by glutathione peroxidase into 12(S)-HETE [75]. Prostaglandin E2 (PGE2), another metabolite of arachidonic acid, has also been shown to induce acute and chronic inflammation and various autoimmune diseases through Th1 differentiation, Th17 cell proliferation, and activation of mast cells [76]. Changes in arachidonic acid metabolism during liver ischemia can trigger the induction of inflammatory injury [77], arachidonic acid metabolism also plays an important role in galactosamine/endotoxin-induced acute liver injury in rats [78]. In this study, the relative expressions of 12(S)-HETE, PGE2, and Alox15 were significantly increased in the Yin_syn_+BGZ group under the intervention of BGZ, while the relative expressions of 12(S)-HETE and PGE2 were significantly decreased in the Yang_syn_+BGZ group, suggesting that the regulation of arachidonic acid metabolism may be another way in which BGZ regulates the effects of liver protection or liver damage in rats with Yang_syn_ and Yin_syn_, respectively.

In the metabolic pathway of porphyrin and chlorophyll, porphyrins mediate related to tissue and cell damage by inducing protein oxidation and aggregation [79], and the liver is usually the source of excessive porphyrin production, porphyrin accumulation induced slight abnormalities of liver function and even liver failure is also a common type of tissue damage [80]. 5-aminolevulinate, the direct metabolite of glycine, can be used as a precursor of heme and participate in the regulation of the production of heme and its metabolite biliverdin [81]. Biliverdin has a good anti-inflammatory effect, can inhibit the expression of toll-like receptor 4 and nitric oxide, and reduces the inflammatory induction of lipopolysaccharide on macrophages [82]. It is used in the protection against various diseases (vascular injury, organ transplantation, etc.). As such, biliverdin can better reduce the ischemia-reperfusion injury in pig liver and have a liver protective effect [83]. Biliverdin reductase B (Blvrb) is a non-redundant nicotinamide adenine dinucleotide (phosphate)-dependent biliverdin reductase that regulates the cellular redox state by converting biliverdin to bilirubin. Its redox function also reduces intracellular reactive oxygen species accumulation [84] and maintains essential cytoprotective functions in recovery from hematopoietic stress [85]. Bilirubin is the main pigment in human bile and the main metabolite of iron porphyrin compounds in the body with certain damage to the brain and nerves [86]. The increase in its level can also lead to impaired liver function, so it is also used as a test for one of the common indicators of jaundice [87].

Blvrb has also been shown to catalyze the reduction of flavin mononucleotide (FMN, also known as Riboflavin-5-phosphate), flavin adenine dinucleotide (FAD), and riboflavin [88], and riboflavin is involved in tyrosine metabolism by sensitizing tyrosine photooxidation [89]. As an essential vitamin, riboflavin, especially its important derivatives FMN and FAD, disruption of riboflavin homeostasis were found to result in multiple systemic dysfunctions, including neuromuscular disease, anemia, fetal dysplasia, and cardiovascular disease [90]. For example, riboflavin (vitamin B2) deficiency can induce oxidative stress, which mediates the occurrence and development of liver injury and intestinal inflammation [91]. Riboflavin can also improve cardiac injury by inhibiting the expression of LDH induced by cardiac reoxygenation [92]. In addition, recent research demonstrates that tyrosine is abnormally elevated in patients with hepatitis, biliary obstruction, or cirrhosis [93], and tyrosine kinase inhibitors can ameliorate liver injury induced by lipopolysaccharide and ischemia-reperfusion via inhibiting inflammation response [94, 95]. Small molecule multi-tyrosine kinase inhibitors were also found could induced severe liver injury in the treatment of tumors [96]. In the study, BGZ significantly increased the expressions of biliverdin and FMN in Yang_syn_ rats accompanied by the inhibition expressions of bilirubin and FMN in Yin_syn_ rats, suggesting that the regulation of porphyrin and chlorophyll metabolism and tyrosine metabolism via Blvrb, may be a mechanism by which BGZ exerts hepatoprotective or liver damage effects on Yang_syn_ and Yin_syn_ rats, respectively.

In the metabolic pathway of tryptophan, tryptophan is involved in immune regulation, neural function, and intestinal homeostasis through the kynurenine pathway for metabolism [97]. For example, kynurenine is an endothelium-derived relaxation factor in the inflammatory process [98] and is metabolized to neurotoxic 3-Hydroxy-L-kynurenine under the action of kynureninase (Kynu), which is involved in the inflammatory process in psoriasis and other inflammatory diseases [99]. Tryptophan metabolism induces aromatic receptor activation and improves alcohol-induced liver injury [100], and supplementing tryptophan could also protect against CCl4-induced liver injury in rats by inhibiting LDH release [101]. Therefore, the imbalance of tryptophan metabolism in diseases ranging from cancer to neurodegenerative diseases has become a research hotspot for the therapeutic targeting of the kynurenine pathway [102]. In this study, compared with the Yang_syn_ group and the Yin_syn_ group, the levels of 3-Hydroxy-L-kynurenine were significantly decreased and increased under the intervention of BGZ, respectively, suggesting the kynurenine metabolism pathway in regulating tryptophan metabolism may be another mechanism by which BGZ exerts hepatoprotective or liver damage effects on Yang_syn_ and Yin_syn_ rats.

Glycine, serine and threonine metabolism are directly or indirectly involved in the above metabolic pathways (arachidonic acid metabolism, sphingolipid metabolism, porphyrin and chlorophyll metabolism) by providing raw materials [103–107]. Glycine, playing important roles in metabolic regulation, antioxidant response, and neurological function, has been widely used to prevent tissue damage, antioxidant and diabetes, obesity, ischemia-reperfusion injury, tumor, and other inflammatory diseases [108]. The decrease in serine level is closely related to the formation of fatty liver [109], and supplementing serine could alleviate alcoholic fatty liver by regulating homocysteine metabolism and adipogenesis [110]. Threonine can reduce chromium-induced oxidative stress and inflammation by activating PI3K/AKT-related signaling pathways, thereby alleviating liver cell damage [111].

The above-discussed pathways changed by BGZ in Yin_syn_ and (or) Yang_syn_, were almost all related to energy metabolism, amino acid metabolism, and lipid metabolism, and the disorder of the above pathways has also been reported in Yin_syn_ and Yang_syn_ [112, 113]. The effects of BGZ on the metabolism of the above pathways have also been reported by Xu et al. [114]. cAMP, the potential biomarkers for Yin/Yang disharmony in TCM [115], can cross-activate protein kinases related to tissue biosynthesis and metabolism (including carbohydrates, lipids, amino acids, cofactors, and vitamins) as cyclic nucleotide effectors [116–118]. LDH and Na^+^-K^+^-ATP_ase_, two indexes directly reflecting energy level, were significantly correlated with cAMP expression [119, 120]. Considering that BGZ can enhance the expression of cAMP-responsive element modulator-τ [121], and the opposite effect of BGZ on cAMP and LDH and the same adjustment trend on Na^+^-K^+^-ATP_ase_, in rats with Yin_syn_ and Yang_syn_, respectively. It is suggested that the cAMP signaling pathway may be one of the mechanisms by which BGZ regulates energy metabolism to affect rats with Yin_syn_ or Yang_syn_. As for the different intervention effects of BGZ on the different syndromes, the interaction of endogenous metabolites and genes may be the main reason, which is worthy of further study in the future.

In conclusion, this study found that BGZ has a double-edged sword-like effect that not only exerts a good hepatoprotective effect on Yang_syn_ rats but also has a potential risk of inducing liver injury in Yin_syn_ rats. The mechanism is mainly reflected in BGZ having different regulatory effects on amino acid metabolism, energy metabolism, lipid metabolism, and metabolism of cofactors and vitamins in the above two different syndromes. In response to the seemingly contradictory results that BGZ has both liver-damaging and liver-protective effects in previous reports [6, 19], this study found that BGZ may have both liver-damaging and liver-protecting effects on predisposed individuals from the perspective of TCM syndrome theory. The study screened out the endogenous markers that can characterize the corresponding predisposed individuals, which will provide a certain reference for the safe and rational application of TCM.

### Supplementary information

** Additional file 1:** **Table S1** Primer sequences of target genes. **Table S2** DEGs and correspondingfold changes (FC) regulated by BGZ in the treatment of Yang_syn_ inrats. **Table S3** DEGs and correspondingfold changes regulated by BGZ in the treatment of Yin_syn_ in rats. **Table S4 **The relationship between the relativeabundance ofDEGsand the levels of serum biochemistry (ALT and AST). **Table S5 **GO functional enrichmentpathways analysis of DEGs regulated by BGZ in Yang_syn_+BGZ ratscompared with Yang_syn_ rats (Padjust < 0.05). **Table S6 **The relationship between the relativeabundance of metabolites and the levels of serum biochemistry(ALT and AST). **Table S7 **Metabolic pathways of BGZin the treatment of Yin_syn_ in rats. **Table S8 **Metabolic pathways of BGZin the treatment of Yang_syn_ in rats. **Figure S1 **ROC analysis of DEGs. **A** the DEGs between the Yang_syn_+BGZ group and the Yang_syn_group; **B** the DEGs between the Yin_syn_+BGZ group and the Yin_syn_group. **Figure S2** **A** The 100-permutationtest for the Yang_syn_+BGZ group and Yang_syn_ group in ESI-mode; **B** The 100-permutation test for Yin_syn_+BGZ group and Yin_syn_group in ESI- mode; **C** The 100-permutation test for Yang_syn_+BGZ groupand Yang_syn_ group in ESI+ mode; **D** The 100-permutation test for Yin_syn_+BGZgroup and Yin_syn_ group in ESI+ mode. **Figure S3** Secondaryfragment ions characteristic maps of identified metabolites. **Figure S4 **ROC analysis ofmetabolites. **A** the metabolites between the Yang_syn_+BGZ group and theYang_syn_ group; **B** the metabolites between the Yin_syn_+BGZgroup and the Yin_syn_ group.
