# Supplementary material for: Yin/Yang associated differential responses to Psoralea corylifolia Linn. In rat models: an integrated metabolomics and transcriptomics study
Source: Chin Med. 2023 Aug 17;18:102. doi: 10.1186/s13020-023-00793-x (PMC10433582; doi:10.1186/s13020-023-00793-x)
Supplement: Supplementary file 1 — Additional file 1: Table S1 Primer sequences of target genes. Table S2 DEGs and correspondingfold changes (FC) regulated by BGZ in the treatment of Yangsyn inrats. Table S3 DEGs and correspondingfold changes regulated by BGZ in the treatment of Yinsyn in rats. Table S4 The relationship between the relativeabundance ofDEGsand the levels of serum biochemistry (ALT and AST). Table S5 GO functional enrichmentpathways analysis of DEGs regulated by BGZ in Yangsyn+BGZ ratscompared with Yangsyn rats (Padjust < 0.05). Table S6 The relationship between the relativeabundance of metabolites and the levels of serum biochemistry(ALT and AST). Table S7 Metabolic pathways of BGZin the treatment of Yinsyn in rats. Table S8 Metabolic pathways of BGZin the treatment of Yangsyn in rats. Figure S1 ROC analysis of DEGs. A the DEGs between the Yangsyn+BGZ group and the Yangsyngroup; B the DEGs between the Yinsyn+BGZ group and the Yinsyngroup. Figure S2 A The 100-permutationtest for the Yangsyn+BGZ group and Yangsyn group in ESI-mode; B The 100-permutation test for Yinsyn+BGZ group and Yinsyngroup in ESI- mode; C The 100-permutation test for Yangsyn+BGZ groupand Yangsyn group in ESI+ mode; D The 100-permutation test for Yinsyn+BGZgroup and Yinsyn group in ESI+ mode. Figure S3 Secondaryfragment ions characteristic maps of identified metabolites. Figure S4 ROC analysis ofmetabolites. A the metabolites between the Yangsyn+BGZ group and theYangsyn group; B the metabolites between the Yinsyn+BGZgroup and the Yinsyn group. [file 13020_2023_793_MOESM1_ESM.docx]

**Supplementary Tables and Figures.**

**Supplemental Table 1** Primer sequences of target genes.

| **Number** | **Genes** | **Primer sequence (5′–3′)** |
| --- | --- | --- |
| 1 | *Aldh1b1_F* | TCGGATTTAGGAGGCTGCAT |
|  | *Aldh1b1_R* | TGACAACCACGGTATTCCCA |
| 2 | *Lgals5_F* | CATACCCGAACCTAGCTGTACC |
|  | *Lgals5_R* | GAGTGTTTCGGACCACAGCA |
| 3 | *Slec25a25_F* | ACCCGGCATTCCCTACTCTT |
|  | *Slec25a25_R* | CTGCTTGCACAATTTTCTGCTTC |
| 4 | *Pim3_F* | ACTGACTTTGATGGCACCCG |
|  | *Pim3_R* | GGAATGTCCCCACACACCAT |
| 5 | *Oaf _F* | TCGCGGATTTCAAGAAGGATG |
|  | *Oaf _R* | CTGGAACTGACTTTGCCCCT |
| 6 | *GAPDH_F* | TCTCTGCTCCTCCCTGTTCT |
|  | *GAPDH_R* | CCGATACGGCCAAATCCGTT |
|  |  |  |

**Supplemental Table 2** DEGs and corresponding fold changes (FC) regulated by BGZ in the treatment of Yang_syn_ in rats.

| **Gene_id** | **Gene name** | **Gene description** | **Log_2_(FC)** | **Padjust** |
| --- | --- | --- | --- | --- |
| ENSRNOG00000026672 | MGC94199 | similar to RIKEN cDNA 2610301B20; EST AI428449 | 7.03 | 0.00 |
| ENSRNOG00000028235 | RGD1560795 | similar to Sepiapterin reductase (SPR) | 6.82 | 0.00 |
| ENSRNOG00000023476 | Slc16a5 | solute carrier family 16 member 5 | 5.60 | 0.01 |
| ENSRNOG00000003280 | Grin2c | glutamate ionotropic receptor NMDA type subunit 2C | 5.36 | 0.00 |
| ENSRNOG00000031207 | LOC500035 | hypothetical protein LOC500035 | 5.04 | 0.03 |
| ENSRNOG00000019500 | Cyp1a1 | cytochrome P450, family 1, subfamily a, polypeptide 1 | 4.93 | 0.00 |
| ENSRNOG00000029588 | LOC100364769 | LRRG00136-like | 4.91 | 0.02 |
| ENSRNOG00000002331 | Aldh3a1 | aldehyde dehydrogenase 3 family, member A1 | 3.98 | 0.00 |
| ENSRNOG00000016173 | Cyp1a2 | cytochrome P450, family 1, subfamily a, polypeptide 2 | 3.58 | 0.00 |
| ENSRNOG00000012772 | Nqo1 | NAD(P)H quinone dehydrogenase 1 | 3.42 | 0.00 |
| ENSRNOG00000000521 | Cdkn1a | cyclin-dependent kinase inhibitor 1A | 2.73 | 0.00 |
| ENSRNOG00000033680 | Cyp2b1 | cytochrome P450, family 2, subfamily b, polypeptide 1 | 2.66 | 0.00 |
| ENSRNOG00000056847 | Gsta3 | glutathione S-transferase alpha 3 | 2.54 | 0.00 |
| ENSRNOG00000011042 | RGD1560925 | similar to 2610034M16Rik protein | 2.50 | 0.01 |
| ENSRNOG00000017899 | Akr7a3 | aldo-keto reductase family 7 member A3 | 2.50 | 0.00 |
| ENSRNOG00000058847 | AABR07044001.4 |  | 2.45 | 0.00 |
| ENSRNOG00000001242 | Gstt3 | glutathione S-transferase, theta 3 | 2.27 | 0.00 |
| ENSRNOG00000000836 | Ltb | lymphotoxin beta | 2.25 | 0.03 |
| ENSRNOG00000019058 | Gstm3l | glutathione S-transferase mu 3-like | 2.25 | 0.01 |
| ENSRNOG00000017445 | Tubb2b | tubulin, beta 2B class IIb | 2.12 | 0.00 |
| ENSRNOG00000043280 | Vom2r37 | vomeronasal 2 receptor, 37 | 1.89 | 0.01 |
| ENSRNOG00000007136 | Anxa7 | annexin A7 | 1.85 | 0.00 |
| ENSRNOG00000006622 | Cry1 | cryptochrome circadian regulator 1 | 1.78 | 0.00 |
| ENSRNOG00000011300 | AABR07031193.1 | laminin subunit alpha 3 | 1.76 | 0.00 |
| ENSRNOG00000013408 | Npas2 | neuronal PAS domain protein 2 | 1.71 | 0.00 |
| ENSRNOG00000017914 | Cavin3 | caveolae associated protein 3 | 1.58 | 0.01 |
| ENSRNOG00000019657 | Mk1 | Mk1 protein | 1.54 | 0.00 |
| ENSRNOG00000056076 | LOC103694877 | macrophage migration inhibitory factor | 1.49 | 0.01 |
| ENSRNOG00000019412 | Rhbg | Rh family B glycoprotein | 1.42 | 0.00 |
| ENSRNOG00000014948 | Osgin1 | oxidative stress induced growth inhibitor 1 | 1.30 | 0.00 |
| ENSRNOG00000012123 | Fdx1 | ferredoxin 1 | 1.28 | 0.04 |
| ENSRNOG00000017558 | Tubb2a | tubulin, beta 2A class IIa | 1.28 | 0.00 |
| ENSRNOG00000003674 | Pir | pirin | 1.27 | 0.00 |
| ENSRNOG00000048682 | Zwint | ZW10 interacting kinetochore protein | 1.26 | 0.02 |
| ENSRNOG00000006589 | Mif | macrophage migration inhibitory factor | 1.23 | 0.00 |
| ENSRNOG00000057832 | Rnf125 | ring finger protein 125 | 1.19 | 0.00 |
| ENSRNOG00000008519 | Dipk2a | divergent protein kinase domain 2A | 1.18 | 0.04 |
| ENSRNOG00000012387 | Glyatl2 | glycine-N-acyltransferase-like 2 | 1.13 | 0.01 |
| ENSRNOG00000024410 | Blvrb | biliverdin reductase B | 0.88 | 0.00 |
| ENSRNOG00000055984 | Adrm1 | adhesion regulating molecule 1 | 0.87 | 0.01 |
| ENSRNOG00000029993 | Kynu | kynureninase | 0.84 | 0.01 |
| ENSRNOG00000015109 | Ubxn8 | UBX domain protein 8 | 0.83 | 0.01 |
| ENSRNOG00000015519 | Ces1d | carboxylesterase 1D | 0.83 | 0.00 |
| ENSRNOG00000009719 | C2cd2l | C2CD2-like | -0.75 | 0.03 |
| ENSRNOG00000036693 | Slc25a10 | solute carrier family 25 member 10 | -0.79 | 0.00 |
| ENSRNOG00000007529 | Bmf | Bcl2 modifying factor | -0.82 | 0.03 |
| ENSRNOG00000003235 | Mgat4b | alpha-1,3-mannosyl-glycoprotein 4-beta-N-acetylglucosaminyltransferase B | -0.84 | 0.01 |
| ENSRNOG00000001189 | Sik1 | salt-inducible kinase 1 | -0.98 | 0.01 |
| ENSRNOG00000001030 | Tsc22d1 | TSC22 domain family, member 1 | -1.01 | 0.00 |
| ENSRNOG00000020194 | Hes6 | hes family bHLH transcription factor 6 | -1.02 | 0.01 |
| ENSRNOG00000033573 | Sgk2 | serum/glucocorticoid regulated kinase 2 | -1.06 | 0.02 |
| ENSRNOG00000018903 | Pik3r1 | phosphoinositide-3-kinase regulatory subunit 1 | -1.07 | 0.05 |
| ENSRNOG00000014876 | Lpin2 | lipin 2 | -1.07 | 0.00 |
| ENSRNOG00000006305 | Slc38a2 | solute carrier family 38, member 2 | -1.15 | 0.00 |
| ENSRNOG00000011361 | Slc37a4 | solute carrier family 37 member 4 | -1.19 | 0.04 |
| ENSRNOG00000009243 | Oaf | out at first homolog | -1.20 | 0.00 |
| ENSRNOG00000029698 | Pim3 | Pim-3 proto-oncogene, serine/threonine kinase | -1.20 | 0.03 |
| ENSRNOG00000020811 | Il6r | interleukin 6 receptor | -1.21 | 0.03 |
| ENSRNOG00000006019 | G0s2 | G0/G1switch 2 | -1.22 | 0.01 |
| ENSRNOG00000061821 | AC109891.1 |  | -1.27 | 0.00 |
| ENSRNOG00000047005 | Kcnk5 | potassium two pore domain channel subfamily K member 5 | -1.28 | 0.03 |
| ENSRNOG00000003977 | Dusp1 | dual specificity phosphatase 1 | -1.29 | 0.00 |
| ENSRNOG00000006118 | Klf10 | Kruppel-like factor 10 | -1.31 | 0.00 |
| ENSRNOG00000011474 | Ppp1r3b | protein phosphatase 1, regulatory subunit 3B | -1.33 | 0.01 |
| ENSRNOG00000017808 | Klf15 | Kruppel-like factor 15 | -1.33 | 0.00 |
| ENSRNOG00000033433 | Csrnp1 | cysteine and serine rich nuclear protein 1 | -1.35 | 0.01 |
| ENSRNOG00000019206 | Nupr1 | nuclear protein 1, transcriptional regulator | -1.36 | 0.00 |
| ENSRNOG00000007601 | Inhbe | inhibin subunit beta E | -1.38 | 0.00 |
| ENSRNOG00000016776 | Extl1 | exostosin-like glycosyltransferase 1 | -1.44 | 0.01 |
| ENSRNOG00000016348 | Tat | tyrosine aminotransferase | -1.45 | 0.00 |
| ENSRNOG00000003463 | Srebf1 | sterol regulatory element binding transcription factor 1 | -1.47 | 0.02 |
| ENSRNOG00000004171 | Dnah9 | dynein, axonemal, heavy chain 9 | -1.53 | 0.02 |
| ENSRNOG00000016037 | Mafb | MAF bZIP transcription factor B | -1.58 | 0.00 |
| ENSRNOG00000020444 | Hcn3 | hyperpolarization-activated cyclic nucleotide-gated potassium channel 3 | -1.62 | 0.01 |
| ENSRNOG00000058186 | Errfi1 | ERBB receptor feedback inhibitor 1 | -1.67 | 0.00 |
| ENSRNOG00000047977 | Tcim | transcriptional and immune response regulator | -1.70 | 0.00 |
| ENSRNOG00000046912 | Nr1d2 | nuclear receptor subfamily 1, group D, member 2 | -1.77 | 0.00 |
| ENSRNOG00000011420 | Mtmr7 | myotubularin related protein 7 | -1.80 | 0.00 |
| ENSRNOG00000009979 | Sebox | SEBOX homeobox | -1.82 | 0.00 |
| ENSRNOG00000018796 | Herpud1 | homocysteine inducible ER protein with ubiquitin like domain 1 | -1.86 | 0.00 |
| ENSRNOG00000020035 | Cyp17a1 | cytochrome P450, family 17, subfamily a, polypeptide 1 | -1.88 | 0.00 |
| ENSRNOG00000007387 | Per1 | period circadian regulator 1 | -1.90 | 0.03 |
| ENSRNOG00000037782 | Gkn3 | gastrokine 3 | -1.96 | 0.03 |
| ENSRNOG00000020254 | Per2 | period circadian regulator 2 | -2.03 | 0.00 |
| ENSRNOG00000010799 | Noct | nocturnin | -2.15 | 0.01 |
| ENSRNOG00000011497 | Aldh1b1 | aldehyde dehydrogenase 1 family, member B1 | -2.16 | 0.00 |
| ENSRNOG00000007229 | Nr0b2 | nuclear receptor subfamily 0, group B, member 2 | -2.16 | 0.03 |
| ENSRNOG00000017693 | Slc2a5 | solute carrier family 2 member 5 | -2.19 | 0.04 |
| ENSRNOG00000013982 | Hsd17b2 | hydroxysteroid (17-beta) dehydrogenase 2 | -2.42 | 0.00 |
| ENSRNOG00000009329 | Nr1d1 | nuclear receptor subfamily 1, group D, member 1 | -2.44 | 0.00 |
| ENSRNOG00000014338 | Slc25a25 | solute carrier family 25 member 25 | -2.46 | 0.00 |
| ENSRNOG00000012404 | Thrsp | thyroid hormone responsive | -2.59 | 0.00 |
| ENSRNOG00000011016 | Slc7a2 | solute carrier family 7 member 2 | -2.60 | 0.01 |
| ENSRNOG00000014008 | Mfsd2a | major facilitator superfamily domain containing 2A | -2.71 | 0.00 |
| ENSRNOG00000001388 | Sds | serine dehydratase | -3.06 | 0.00 |
| ENSRNOG00000003634 | Zfp354a | zinc finger protein 354A | -3.10 | 0.00 |
| ENSRNOG00000018413 | Per3 | period circadian regulator 3 | -3.31 | 0.00 |
| ENSRNOG00000001113 | Mmd2 | monocyte to macrophage differentiation-associated 2 | -3.46 | 0.00 |
| ENSRNOG00000021027 | Dbp | D-box binding PAR bZIP transcription factor | -3.95 | 0.00 |
| ENSRNOG00000018448 | Cend1 | cell cycle exit and neuronal differentiation 1 | -4.49 | 0.00 |
| ENSRNOG00000040242 | Epm2a | EPM2A glucan phosphatase, laforin | -5.11 | 0.00 |
| ENSRNOG00000029042 | Mt-nd6 | mitochondrially encoded NADH dehydrogenase 6 | -25.63 | 0.00 |

**Supplemental Table 3** DEGs and corresponding fold changes regulated by BGZ in the treatment of Yin_syn_ in rats.

| **Gene_id** | **Gene name** | **Gene description** | **Log_2_(FC)** | **Padjust** |
| --- | --- | --- | --- | --- |
| ENSRNOG00000019183 | Alox15 | arachidonate 15-lipoxygenase | 3.55 | 0.00 |
| ENSRNOG00000019500 | Cyp1a1 | cytochrome P450, family 1, subfamily a, polypeptide 1 | 2.92 | 0.00 |
| ENSRNOG00000009481 | Ddhd1 | DDHD domain containing 1 | 2.39 | 0.00 |
| ENSRNOG00000055765 | Rasl10b | RAS-like, family 10, member B | 2.04 | 0.02 |
| ENSRNOG00000020165 | Ahsp | alpha hemoglobin stabilizing protein | 1.97 | 0.03 |
| ENSRNOG00000058105 | Hbb | hemoglobin subunit beta | 1.86 | 0.00 |
| ENSRNOG00000000167 | Alas2 | 5'-aminolevulinate synthase 2 | 1.72 | 0.00 |
| ENSRNOG00000029886 | Hba-a1 | hemoglobin alpha, adult chain 1 | 1.65 | 0.00 |
| ENSRNOG00000047321 | Hba-a2 | hemoglobin alpha, adult chain 2 | 1.58 | 0.00 |
| ENSRNOG00000012557 | Lgals5 | lectin, galactose binding, soluble 5 | 1.58 | 0.01 |
| ENSRNOG00000047098 | Hbb-bs | hemoglobin, beta adult s chain | 1.51 | 0.00 |
| ENSRNOG00000061299 | LOC100134871 | beta globin minor gene | 1.31 | 0.00 |
| ENSRNOG00000009712 | Gale | UDP-galactose-4-epimerase | 1.30 | 0.01 |
| ENSRNOG00000060629 | LOC103694855 | hemoglobin subunit beta-2-like | 1.21 | 0.03 |
| ENSRNOG00000010133 | Bpgm | bisphosphoglycerate mutase | 1.07 | 0.03 |
| ENSRNOG00000011276 | Tmem254 | transmembrane protein 254 | 0.95 | 0.05 |
| ENSRNOG00000005480 | Ybx3 | Y box binding protein 3 | 0.82 | 0.00 |
| ENSRNOG00000014613 | Ddah1 | dimethylarginine dimethylaminohydrolase 1 | -0.74 | 0.01 |
| ENSRNOG00000017840 | Pik3c3 | phosphatidylinositol 3-kinase, catalytic subunit type 3 | -0.88 | 0.03 |
| ENSRNOG00000060410 | Pcdh1 | protocadherin 1 | -0.95 | 0.02 |
| ENSRNOG00000053850 | Rdh5 | retinol dehydrogenase 5 | -1.20 | 0.01 |
| ENSRNOG00000002210 | Hsd17b11 | hydroxysteroid (17-beta) dehydrogenase 11 | -1.28 | 0.03 |
| ENSRNOG00000029651 | LOC100365958 | Rdh2 protein-like | -1.39 | 0.03 |
| ENSRNOG00000010714 | Fgl1 | fibrinogen-like 1 | -1.47 | 0.02 |
| ENSRNOG00000019058 | Gstm3l | glutathione S-transferase mu 3-like | -2.57 | 0.05 |
| ENSRNOG00000016957 | Igfbp2 | insulin-like growth factor binding protein 2 | -2.72 | 0.00 |
| ENSRNOG00000007012 | Ly75 | lymphocyte antigen 75 | -2.76 | 0.01 |
| ENSRNOG00000046643 | Cyp3a9 | cytochrome P450, family 3, subfamily a, polypeptide 9 | -3.21 | 0.01 |

**Supplementary Table 4** The relationship between the relative abundance of DEGs and the levels of serum biochemistry (ALT and AST).

| **Gene** | **ALT** | | **AST** | | **Gene** | **ALT** | | **AST** | | **Gene** | **ALT** | | **AST** | | **Gene** | **ALT** | | **AST** | |
| --- | --- | --- | --- | --- | --- | --- | --- | --- | --- | --- | --- | --- | --- | --- | --- | --- | --- | --- | --- |
|  | **r** | **P value** | **r** | **P value** |  | **r** | **P value** | **r** | **P value** |  | **r** | **P value** | **r** | **P value** |  | **r** | **P value** | **r** | **P value** |
| Sebox | 0.37 | 0.07 | 0.59 | 0.00 | Nr1d2 | 0.17 | 0.42 | 0.33 | 0.11 | Hsd17b11 | 0.05 | 0.82 | 0.25 | 0.23 | Dbp | 0.09 | 0.69 | 0.14 | 0.51 |
| Hcn3 | 0.44 | 0.03 | 0.49 | 0.01 | Ahsp | 0.62 | 0.00 | 0.33 | 0.11 | Tubb2a | 0.24 | 0.25 | 0.25 | 0.23 | Mif | -0.03 | 0.90 | 0.13 | 0.52 |
| LOC100364769 | -0.41 | 0.04 | -0.49 | 0.01 | LOC103694855 | 0.56 | 0.00 | 0.32 | 0.11 | Alas2 | 0.54 | 0.01 | 0.25 | 0.24 | Pir | -0.03 | 0.89 | 0.13 | 0.53 |
| Cyp1a1 | -0.04 | 0.87 | -0.49 | 0.01 | Klf15 | 0.26 | 0.21 | 0.32 | 0.11 | Klf10 | 0.23 | 0.26 | 0.24 | 0.24 | Gale | 0.49 | 0.01 | 0.12 | 0.55 |
| Mmd2 | 0.33 | 0.11 | 0.47 | 0.02 | Cavin3 | 0.34 | 0.10 | 0.32 | 0.12 | Sds | 0.06 | 0.79 | 0.24 | 0.24 | Fdx1 | 0.07 | 0.74 | 0.12 | 0.57 |
| Aldh1b1 | 0.59 | 0.00 | 0.46 | 0.02 | Hbb-bs | 0.61 | 0.00 | 0.32 | 0.12 | Tcim | 0.38 | 0.06 | 0.24 | 0.25 | Tmem254 | 0.45 | 0.03 | 0.11 | 0.60 |
| LOC500035 | -0.22 | 0.28 | -0.46 | 0.02 | Kynu | 0.32 | 0.12 | 0.32 | 0.12 | Lpin2 | 0.30 | 0.14 | 0.24 | 0.25 | Zwint | 0.04 | 0.84 | 0.11 | 0.60 |
| Lgals5 | 0.63 | 0.00 | 0.45 | 0.02 | MGC94199 | -0.03 | 0.90 | -0.32 | 0.12 | Il6r | 0.31 | 0.13 | 0.22 | 0.28 | RGD1560925 | -0.37 | 0.07 | 0.10 | 0.62 |
| Mtmr7 | 0.33 | 0.11 | 0.44 | 0.03 | Srebf1 | 0.48 | 0.02 | 0.31 | 0.13 | Rhbg | -0.06 | 0.79 | -0.22 | 0.30 | Ltb | 0.04 | 0.84 | -0.10 | 0.64 |
| Slc25a25 | 0.50 | 0.01 | 0.42 | 0.03 | Ybx3 | 0.46 | 0.02 | 0.31 | 0.13 | Ppp1r3b | 0.43 | 0.03 | 0.21 | 0.30 | Slc2a5 | 0.18 | 0.39 | 0.10 | 0.64 |
| Pim3 | 0.53 | 0.01 | 0.41 | 0.04 | Slc16a5 | -0.39 | 0.05 | -0.31 | 0.13 | Dipk2a | 0.20 | 0.34 | 0.21 | 0.31 | Alox15 | 0.46 | 0.02 | 0.09 | 0.65 |
| Oaf | 0.52 | 0.01 | 0.41 | 0.04 | Errfi1 | 0.41 | 0.04 | 0.31 | 0.14 | Per1 | 0.29 | 0.16 | 0.20 | 0.34 | Cyp17a1 | 0.00 | 1.00 | 0.09 | 0.66 |
| Bpgm | 0.57 | 0.00 | 0.39 | 0.05 | Pcdh1 | 0.28 | 0.17 | 0.30 | 0.14 | Ly75 | -0.30 | 0.14 | 0.20 | 0.35 | Nqo1 | 0.04 | 0.87 | -0.08 | 0.70 |
| Tat | 0.47 | 0.02 | 0.39 | 0.05 | Aldh3a1 | -0.38 | 0.06 | -0.30 | 0.14 | Fgl1 | 0.10 | 0.62 | 0.19 | 0.36 | Gstt3 | 0.13 | 0.55 | 0.08 | 0.71 |
| RGD1560795 | 0.05 | 0.81 | -0.39 | 0.05 | Nr1d1 | 0.15 | 0.47 | 0.30 | 0.14 | Glyatl2 | 0.00 | 0.99 | 0.19 | 0.37 | G0s2 | 0.21 | 0.32 | 0.08 | 0.71 |
| Sgk2 | 0.66 | 0.00 | 0.39 | 0.06 | Herpud1 | 0.45 | 0.03 | 0.30 | 0.14 | AC109891.1 | 0.25 | 0.22 | 0.18 | 0.38 | Zfp354a | 0.16 | 0.44 | 0.07 | 0.73 |
| Thrsp | 0.63 | 0.00 | 0.39 | 0.06 | Hba-a1 | 0.61 | 0.00 | 0.30 | 0.15 | Mafb | 0.17 | 0.42 | 0.18 | 0.38 | Mt-nd6 | 0.31 | 0.14 | -0.07 | 0.73 |
| Kcnk5 | 0.45 | 0.02 | 0.38 | 0.06 | C2cd2l | 0.52 | 0.01 | 0.30 | 0.15 | Mfsd2a | 0.36 | 0.08 | 0.18 | 0.40 | Noct | 0.35 | 0.09 | 0.07 | 0.74 |
| Hes6 | 0.59 | 0.00 | 0.38 | 0.06 | LOC100134871 | 0.56 | 0.00 | 0.29 | 0.15 | Slc38a2 | 0.29 | 0.17 | 0.18 | 0.40 | Pik3r1 | 0.28 | 0.18 | 0.07 | 0.75 |
| Pik3c3 | -0.09 | 0.68 | 0.38 | 0.06 | Slc7a2 | -0.02 | 0.91 | 0.29 | 0.15 | Npas2 | 0.17 | 0.42 | 0.17 | 0.41 | Ces1d | 0.03 | 0.87 | 0.07 | 0.75 |
| Tubb2b | 0.25 | 0.23 | 0.37 | 0.07 | Grin2c | 0.07 | 0.73 | -0.29 | 0.16 | Rnf125 | 0.06 | 0.79 | 0.17 | 0.42 | Ddhd1 | 0.49 | 0.01 | 0.06 | 0.78 |
| Gkn3 | 0.44 | 0.03 | 0.36 | 0.07 | Mgat4b | 0.37 | 0.07 | 0.29 | 0.16 | Cyp3a9 | -0.03 | 0.87 | 0.17 | 0.42 | Akr7a3 | 0.01 | 0.95 | -0.05 | 0.80 |
| Nr0b2 | 0.38 | 0.06 | 0.36 | 0.08 | Per3 | 0.15 | 0.47 | 0.28 | 0.17 | Tsc22d1 | 0.26 | 0.21 | 0.17 | 0.43 | Inhbe | 0.14 | 0.51 | 0.05 | 0.81 |
| Cend1 | 0.38 | 0.06 | 0.36 | 0.08 | AABR07031193.1 | -0.45 | 0.02 | -0.28 | 0.18 | Ubxn8 | -0.28 | 0.18 | -0.16 | 0.43 | Dusp1 | 0.12 | 0.57 | 0.04 | 0.84 |
| Csrnp1 | 0.49 | 0.01 | 0.35 | 0.08 | Slc25a10 | 0.36 | 0.08 | 0.28 | 0.18 | Cyp1a2 | 0.16 | 0.44 | -0.16 | 0.44 | Blvrb | -0.10 | 0.65 | 0.03 | 0.87 |
| Gsta3 | -0.01 | 0.95 | -0.35 | 0.08 | Cry1 | -0.39 | 0.06 | -0.27 | 0.19 | Mk1 | 0.19 | 0.36 | 0.16 | 0.46 | AABR07044001.4 | -0.12 | 0.57 | 0.03 | 0.88 |
| Extl1 | 0.25 | 0.23 | 0.35 | 0.09 | Dnah9 | 0.27 | 0.19 | 0.27 | 0.19 | Ddah1 | 0.06 | 0.79 | 0.16 | 0.46 | Gstm3l | -0.30 | 0.14 | -0.03 | 0.89 |
| Hbb | 0.64 | 0.00 | 0.35 | 0.09 | Hba-a2 | 0.59 | 0.00 | 0.27 | 0.19 | Cdkn1a | 0.20 | 0.34 | 0.15 | 0.49 | Epm2a | -0.06 | 0.79 | -0.03 | 0.89 |
| Slc37a4 | 0.48 | 0.02 | 0.34 | 0.09 | Igfbp2 | 0.11 | 0.60 | 0.26 | 0.20 | Osgin1 | -0.07 | 0.73 | 0.14 | 0.50 | Nupr1 | -0.09 | 0.67 | 0.02 | 0.92 |
| Rdh16 | 0.05 | 0.81 | 0.34 | 0.09 | Vom2r37 | 0.20 | 0.34 | 0.26 | 0.20 | LOC103694877 | -0.05 | 0.81 | 0.14 | 0.50 | Anxa7 | -0.04 | 0.83 | 0.02 | 0.93 |
| Sik1 | 0.22 | 0.29 | 0.34 | 0.09 | Adrm1 | 0.18 | 0.40 | 0.26 | 0.21 | Cyp2b1 | -0.04 | 0.87 | -0.14 | 0.50 | Hsd17b2 | 0.11 | 0.59 | 0.01 | 0.97 |
| Bmf | 0.44 | 0.03 | 0.34 | 0.10 | Rdh5 | 0.12 | 0.57 | 0.25 | 0.22 | Rasl10b | 0.30 | 0.15 | 0.14 | 0.51 | Per2 | 0.06 | 0.78 | 0.01 | 0.97 |

**Supplementary Table 5** GO functional enrichment pathways analysis of DEGs regulated by BGZ in Yang_syn_+BGZ rats compared with Yang_syn_ rats (Padjust < 0.05).

| **GO ID** | **Term Type** | **Description** | ***Padjust*** | **GO ID** | **Term Type** | **Description** | ***Padjust*** |
| --- | --- | --- | --- | --- | --- | --- | --- |
| GO:0032922 | BP | circadian regulation of gene expression | *0.001* | GO:0008202 | BP | steroid metabolic process | *0.021* |
| GO:0042752 | BP | regulation of circadian rhythm | *0.001* | GO:0048523 | BP | negative regulation of cellular process | *0.021* |
| GO:0048511 | BP | rhythmic process | *0.001* | GO:0043227 | CC | membrane-bounded organelle | *0.021* |
| GO:0031667 | BP | response to nutrient levels | *0.001* | GO:1901654 | BP | response to ketone | *0.022* |
| GO:0009991 | BP | response to extracellular stimulus | *0.001* | GO:0042542 | BP | response to hydrogen peroxide | *0.023* |
| GO:1901698 | BP | response to nitrogen compound | *0.001* | GO:0006950 | BP | response to stress | *0.023* |
| GO:0007584 | BP | response to nutrient | *0.001* | GO:1901615 | BP | organic hydroxy compound metabolic process | *0.023* |
| GO:0033993 | BP | response to lipid | *0.001* | GO:0008514 | MF | organic anion transmembrane transporter activity | *0.023* |
| GO:0009892 | BP | negative regulation of metabolic process | *0.001* | GO:0000122 | BP | negative regulation of transcription by RNA polymerase II | *0.024* |
| GO:0010243 | BP | response to organonitrogen compound | *0.001* | GO:0051172 | BP | negative regulation of nitrogen compound metabolic process | *0.025* |
| GO:0007623 | BP | circadian rhythm | *0.001* | GO:0032094 | BP | response to food | *0.025* |
| GO:0014070 | BP | response to organic cyclic compound | *0.001* | GO:1903409 | BP | reactive oxygen species biosynthetic process | *0.025* |
| GO:0010033 | BP | response to organic substance | *0.001* | GO:0031324 | BP | negative regulation of cellular metabolic process | *0.026* |
| GO:0042221 | BP | response to chemical | *0.001* | GO:0003044 | BP | regulation of systemic arterial blood pressure mediated by a chemical signal | *0.028* |
| GO:0043153 | BP | entrainment of circadian clock by photoperiod | *0.001* | GO:0043276 | BP | anoikis | *0.029* |
| GO:1901700 | BP | response to oxygen-containing compound | *0.001* | GO:0018894 | BP | dibenzo-p-dioxin metabolic process | *0.029* |
| GO:0009628 | BP | response to abiotic stimulus | *0.001* | GO:0009314 | BP | response to radiation | *0.029* |
| GO:0009649 | BP | entrainment of circadian clock | *0.002* | GO:1901652 | BP | response to peptide | *0.030* |
| GO:0009605 | BP | response to external stimulus | *0.002* | GO:1903825 | BP | organic acid transmembrane transport | *0.032* |
| GO:0009648 | BP | photoperiodism | *0.002* | GO:1905039 | BP | carboxylic acid transmembrane transport | *0.032* |
| GO:0050896 | BP | response to stimulus | *0.002* | GO:0032501 | BP | multicellular organismal process | *0.033* |
| GO:0009416 | BP | response to light stimulus | *0.003* | GO:0009267 | BP | cellular response to starvation | *0.033* |
| GO:0048545 | BP | response to steroid hormone | *0.003* | GO:0010817 | BP | regulation of hormone levels | *0.034* |
| GO:0051384 | BP | response to glucocorticoid | *0.003* | GO:0065008 | BP | regulation of biological quality | *0.034* |
| GO:0015980 | BP | energy derivation by oxidation of organic compounds | *0.004* | GO:0009056 | BP | catabolic process | *0.034* |
| GO:0010629 | BP | negative regulation of gene expression | *0.004* | GO:0005976 | BP | polysaccharide metabolic process | *0.034* |
| GO:0010605 | BP | negative regulation of macromolecule metabolic process | *0.004* | GO:0044264 | BP | cellular polysaccharide metabolic process | *0.034* |
| GO:0031960 | BP | response to corticosteroid | *0.004* | GO:0048513 | BP | animal organ development | *0.034* |
| GO:0032868 | BP | response to insulin | *0.004* | GO:0046943 | MF | carboxylic acid transmembrane transporter activity | *0.035* |
| GO:0048519 | BP | negative regulation of biological process | *0.006* | GO:0005342 | MF | organic acid transmembrane transporter activity | *0.036* |
| GO:0043231 | CC | intracellular membrane-bounded organelle | *0.006* | GO:0016491 | MF | oxidoreductase activity | *0.037* |
| GO:0032502 | BP | developmental process | *0.007* | GO:0140110 | MF | transcription regulator activity | *0.037* |
| GO:0010866 | BP | regulation of triglyceride biosynthetic process | *0.008* | GO:1901361 | BP | organic cyclic compound catabolic process | *0.038* |
| GO:0032572 | BP | response to menaquinone | *0.009* | GO:0010035 | BP | response to inorganic substance | *0.038* |
| GO:0101021 | MF | estrogen 2-hydroxylase activity | *0.009* | GO:0046683 | BP | response to organophosphorus | *0.039* |
| GO:0009404 | BP | toxin metabolic process | *0.009* | GO:0044262 | BP | cellular carbohydrate metabolic process | *0.039* |
| GO:0007568 | BP | aging | *0.009* | GO:1901701 | BP | cellular response to oxygen-containing compound | *0.041* |
| GO:0009719 | BP | response to endogenous stimulus | *0.010* | GO:0034220 | BP | ion transmembrane transport | *0.041* |
| GO:0006073 | BP | cellular glucan metabolic process | *0.011* | GO:0045934 | BP | negative regulation of nucleobase-containing compound metabolic process | *0.041* |
| GO:0044042 | BP | glucan metabolic process | *0.011* | GO:0043434 | BP | response to peptide hormone | *0.043* |
| GO:0005977 | BP | glycogen metabolic process | *0.011* | GO:0019222 | BP | regulation of metabolic process | *0.044* |
| GO:0033273 | BP | response to vitamin | *0.011* | GO:0005634 | CC | nucleus | *0.044* |
| GO:0044248 | BP | cellular catabolic process | *0.012* | GO:0006778 | BP | porphyrin-containing compound metabolic process | *0.045* |
| GO:0009725 | BP | response to hormone | *0.013* | GO:0051716 | BP | cellular response to stimulus | *0.048* |
| GO:0006357 | BP | regulation of transcription by RNA polymerase II | *0.013* | GO:0010558 | BP | negative regulation of macromolecule biosynthetic process | *0.049* |
| GO:0006112 | BP | energy reserve metabolic process | *0.014* | GO:0005829 | CC | cytosol | *0.049* |
| GO:0051591 | BP | response to cAMP | *0.018* | GO:0101020 | MF | estrogen 16-alpha-hydroxylase activity | *0.049* |
| GO:0097167 | BP | circadian regulation of translation | *0.019* |  |  |  |  |

**Supplementary Table 6** The relationship between the relative abundance of metabolites and the levels of serum biochemistry (ALT and AST).

| **Metabolites** | **ALT** | | **AST** | | **Metabolites** | **ALT** | | **AST** | |
| --- | --- | --- | --- | --- | --- | --- | --- | --- | --- |
|  | **r** | **P** | **r** | **P** |  | **r** | **P** | **r** | **P** |
| Phosphatidate | 0.55 | 0.00 | 0.54 | 0.00 | Lanosterin | -0.62 | 0.00 | -0.43 | 0.01 |
| (R)-4'-Phosphopantothenoyl-L-cysteine | -0.29 | 0.07 | 0.16 | 0.34 | L-Serine | -0.21 | 0.20 | 0.23 | 0.17 |
| 12(S)-HETE | 0.10 | 0.53 | 0.05 | 0.78 | L-Urobilinogen | 0.22 | 0.16 | -0.13 | 0.43 |
| 1-Acyl-sn-glycero-3-phosphocholine | -0.34 | 0.03 | 0.00 | 0.99 | Methylcysteine | -0.12 | 0.45 | -0.07 | 0.67 |
| 2-Methoxyestradiol | -0.30 | 0.06 | -0.02 | 0.89 | Methylglyoxal | -0.41 | 0.01 | -0.15 | 0.36 |
| 3,4-Dihydroxymandelate | 0.09 | 0.57 | -0.31 | 0.06 | Glucaric acid | -0.22 | 0.16 | 0.24 | 0.15 |
| 3-Carboxy-1-hydroxypropylthiamine diphosphate | 0.25 | 0.11 | 0.04 | 0.81 | N6-Acetyl-L-lysine | -0.30 | 0.05 | -0.24 | 0.15 |
| 3-Hydroxy-L-kynurenine | -0.02 | 0.92 | -0.27 | 0.09 | N-Acylsphingosine | -0.53 | 0.00 | -0.43 | 0.01 |
| 5-Aminolevulinate | -0.17 | 0.27 | -0.11 | 0.49 | Phosphatidylcholine | -0.60 | 0.00 | -0.47 | 0.00 |
| 5-Hydroxy-N-formylkynurenine | -0.58 | 0.00 | -0.26 | 0.11 | Phosphatidylethanolamine | 0.08 | 0.62 | 0.01 | 0.95 |
| beta-D-Glucuronoside | -0.58 | 0.00 | -0.35 | 0.03 | Phosphorylcholine | -0.33 | 0.04 | 0.04 | 0.81 |
| Bilirubin | 0.10 | 0.53 | 0.28 | 0.09 | Prostaglandin E2 | -0.15 | 0.33 | -0.41 | 0.01 |
| Biliverdin | -0.61 | 0.00 | -0.36 | 0.03 | Pyridoxal | -0.52 | 0.00 | -0.27 | 0.09 |
| Cholic acid | -0.51 | 0.00 | -0.29 | 0.08 | Riboflavin-5-phosphate | -0.14 | 0.39 | 0.15 | 0.35 |
| D-Glucosamine 6-phosphate | -0.39 | 0.01 | 0.10 | 0.56 | Tetrahydrocorticosterone | -0.35 | 0.02 | 0.01 | 0.95 |
| D-Leucate | -0.58 | 0.00 | -0.42 | 0.01 | Thyroxine | -0.39 | 0.01 | -0.22 | 0.18 |
| Docosapentaenoic acid (22n-3) | -0.69 | 0.00 | -0.32 | 0.05 | Tyramine | -0.15 | 0.35 | -0.31 | 0.06 |
| D-Xylitol | -0.14 | 0.38 | 0.18 | 0.27 | Uracil | 0.49 | 0.00 | 0.50 | 0.00 |
| gamma-Aminobutyric acid | -0.61 | 0.00 | -0.37 | 0.02 | Uridine | 0.26 | 0.10 | -0.02 | 0.91 |
| N1-(5-Phospho-a-D-ribosyl)-5,6-dimethylbenzimidazole | 0.49 | 0.00 | 0.49 | 0.00 | α-Ketoglutarate | -0.05 | 0.74 | 0.12 | 0.47 |

**Supplementary Table 7** Metabolic pathways of BGZ in the treatment of Yin_syn_ in rats.

| **Comparison** | **Pathways** | **Number of metabolites** | **P value** | **Impact** |
| --- | --- | --- | --- | --- |
| Yin_syn_+BGZ vs Yin_syn_ | Pantothenate and CoA biosynthesis | 2 | 0.05 | 0.18 |
| Yin_syn_+BGZ vs Yin_syn_ | Sphingolipid metabolism | 2 | 0.06 | 0.27 |
| Yin_syn_+BGZ vs Yin_syn_ | Riboflavin metabolism | 1 | 0.07 | 0.50 |
| Yin_syn_+BGZ vs Yin_syn_ | Alanine, aspartate and glutamate metabolism | 2 | 0.09 | 0.09 |
| Yin_syn_+BGZ vs Yin_syn_ | Glycine, serine and threonine metabolism | 2 | 0.12 | 0.22 |
| Yin_syn_+BGZ vs Yin_syn_ | Ascorbate and aldarate metabolism | 1 | 0.14 | 0.00 |
| Yin_syn_+BGZ vs Yin_syn_ | Arachidonic acid metabolism | 2 | 0.14 | 0.00 |
| Yin_syn_+BGZ vs Yin_syn_ | Glycerophospholipid metabolism | 2 | 0.14 | 0.15 |
| Yin_syn_+BGZ vs Yin_syn_ | Vitamin B6 metabolism | 1 | 0.16 | 0.49 |
| Yin_syn_+BGZ vs Yin_syn_ | Pyrimidine metabolism | 2 | 0.16 | 0.09 |
| Yin_syn_+BGZ vs Yin_syn_ | Tryptophan metabolism | 2 | 0.18 | 0.11 |
| Yin_syn_+BGZ vs Yin_syn_ | Tyrosine metabolism | 2 | 0.18 | 0.03 |
| Yin_syn_+BGZ vs Yin_syn_ | Butanoate metabolism | 1 | 0.25 | 0.03 |
| Yin_syn_+BGZ vs Yin_syn_ | Glycerolipid metabolism | 1 | 0.26 | 0.01 |
| Yin_syn_+BGZ vs Yin_syn_ | Pentose and glucuronate interconversions | 1 | 0.29 | 0.17 |
| Yin_syn_+BGZ vs Yin_syn_ | Citrate cycle (TCA cycle) | 1 | 0.32 | 0.09 |
| Yin_syn_+BGZ vs Yin_syn_ | beta-Alanine metabolism | 1 | 0.33 | 0.00 |
| Yin_syn_+BGZ vs Yin_syn_ | Pyruvate metabolism | 1 | 0.34 | 0.03 |
| Yin_syn_+BGZ vs Yin_syn_ | Phosphatidylinositol signaling system | 1 | 0.41 | 0.00 |
| Yin_syn_+BGZ vs Yin_syn_ | Porphyrin and chlorophyll metabolism | 1 | 0.44 | 0.05 |
| Yin_syn_+BGZ vs Yin_syn_ | Glyoxylate and dicarboxylate metabolism | 1 | 0.46 | 0.04 |
| Yin_syn_+BGZ vs Yin_syn_ | Cysteine and methionine metabolism | 1 | 0.47 | 0.02 |
| Yin_syn_+BGZ vs Yin_syn_ | Amino sugar and nucleotide sugar metabolism | 1 | 0.51 | 0.03 |
| Yin_syn_+BGZ vs Yin_syn_ | Arginine and proline metabolism | 1 | 0.52 | 0.02 |
| Yin_syn_+BGZ vs Yin_syn_ | Steroid biosynthesis | 1 | 0.55 | 0.06 |
| Yin_syn_+BGZ vs Yin_syn_ | Primary bile acid biosynthesis | 1 | 0.59 | 0.00 |
| Yin_syn_+BGZ vs Yin_syn_ | Aminoacyl-tRNA biosynthesis | 1 | 0.60 | 0.17 |
| Yin_syn_+BGZ vs Yin_syn_ | Purine metabolism | 1 | 0.71 | 0.06 |
| Yin_syn_+BGZ vs Yin_syn_ | Steroid hormone biosynthesis | 1 | 0.81 | 0.01 |

**Supplementary Table 8** Metabolic pathways of BGZ in the treatment of Yang_syn_ in rats.

| **Comparison** | **Pathways** | **Number of metabolites** | **P value** | **Impact** |
| --- | --- | --- | --- | --- |
| Yang_syn_+BGZ vs Yang_syn_ | Glycerophospholipid metabolism | 5 | 0.00 | 0.37 |
| Yang_syn_+BGZ vs Yang_syn_ | Arachidonic acid metabolism | 3 | 0.02 | 0.00 |
| Yang_syn_+BGZ vs Yang_syn_ | Pantothenate and CoA biosynthesis | 2 | 0.04 | 0.18 |
| Yang_syn_+BGZ vs Yang_syn_ | Riboflavin metabolism | 1 | 0.06 | 0.50 |
| Yang_syn_+BGZ vs Yang_syn_ | Alanine, aspartate and glutamate metabolism | 2 | 0.07 | 0.05 |
| Yang_syn_+BGZ vs Yang_syn_ | Linoleic acid metabolism | 1 | 0.08 | 0.00 |
| Yang_syn_+BGZ vs Yang_syn_ | Porphyrin and chlorophyll metabolism | 2 | 0.08 | 0.10 |
| Yang_syn_+BGZ vs Yang_syn_ | D-Glutamine and D-glutamate metabolism | 1 | 0.09 | 0.00 |
| Yang_syn_+BGZ vs Yang_syn_ | Vitamin B6 metabolism | 1 | 0.14 | 0.49 |
| Yang_syn_+BGZ vs Yang_syn_ | Tyrosine metabolism | 2 | 0.15 | 0.02 |
| Yang_syn_+BGZ vs Yang_syn_ | alpha-Linolenic acid metabolism | 1 | 0.19 | 0.00 |
| Yang_syn_+BGZ vs Yang_syn_ | Arginine biosynthesis | 1 | 0.20 | 0.00 |
| Yang_syn_+BGZ vs Yang_syn_ | Glycosylphosphatidylinositol (GPI)-anchor biosynthesis | 1 | 0.20 | 0.00 |
| Yang_syn_+BGZ vs Yang_syn_ | Butanoate metabolism | 1 | 0.22 | 0.00 |
| Yang_syn_+BGZ vs Yang_syn_ | Glycerolipid metabolism | 1 | 0.23 | 0.01 |
| Yang_syn_+BGZ vs Yang_syn_ | Pentose and glucuronate interconversions | 1 | 0.25 | 0.14 |
| Yang_syn_+BGZ vs Yang_syn_ | Citrate cycle (TCA cycle) | 1 | 0.28 | 0.06 |
| Yang_syn_+BGZ vs Yang_syn_ | beta-Alanine metabolism | 1 | 0.29 | 0.00 |
| Yang_syn_+BGZ vs Yang_syn_ | Phosphatidylinositol signaling system | 1 | 0.37 | 0.00 |
| Yang_syn_+BGZ vs Yang_syn_ | Glycine, serine and threonine metabolism | 1 | 0.42 | 0.00 |
| Yang_syn_+BGZ vs Yang_syn_ | Amino sugar and nucleotide sugar metabolism | 1 | 0.46 | 0.03 |
| Yang_syn_+BGZ vs Yang_syn_ | Pyrimidine metabolism | 1 | 0.47 | 0.07 |
| Yang_syn_+BGZ vs Yang_syn_ | Purine metabolism | 1 | 0.66 | 0.06 |
| Yang_syn_+BGZ vs Yang_syn_ | Steroid hormone biosynthesis | 1 | 0.76 | 0.00 |


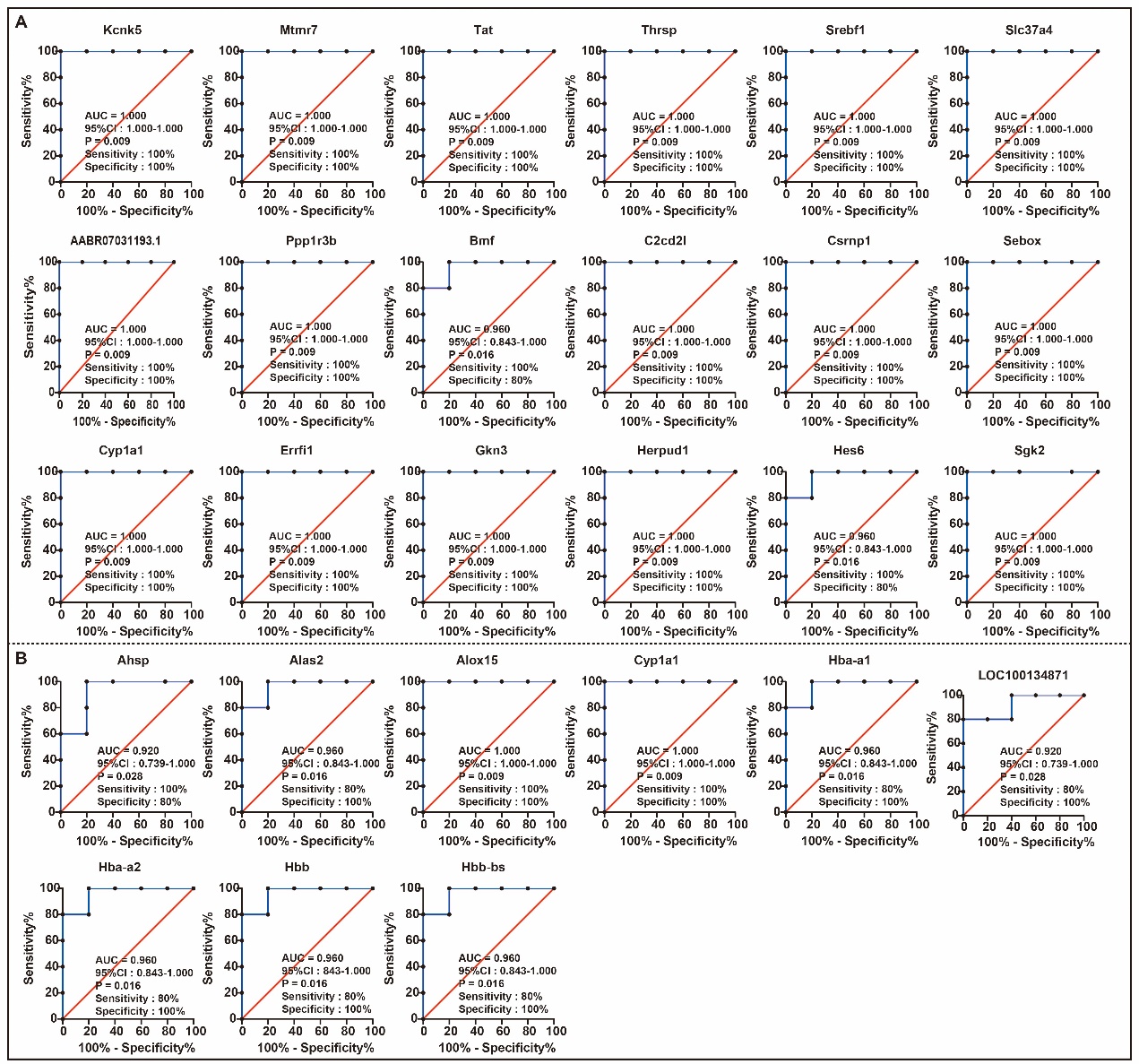


**Supplementary Figure 1** ROC analysis of DEGs. (A) the DEGs between the Yang_syn_+BGZ group and the Yang_syn_ group; (B) the DEGs between the Yin_syn_+BGZ group and the Yin_syn_ group.


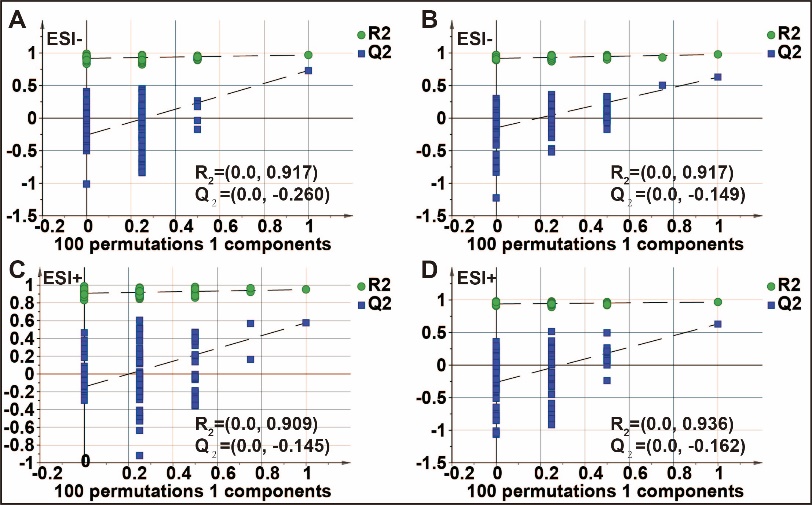


**Supplementary Figure 2** (A) The 100-permutation test for the Yang_syn_+BGZ group and Yang_syn_ group in ESI- mode; (B) The 100-permutation test for Yin_syn_+BGZ group and Yin_syn_ group in ESI- mode; (C) The 100-permutation test for Yang_syn_+BGZ group and Yang_syn_ group in ESI+ mode; (D) The 100-permutation test for Yin_syn_+BGZ group and Yin_syn_ group in ESI+ mode.


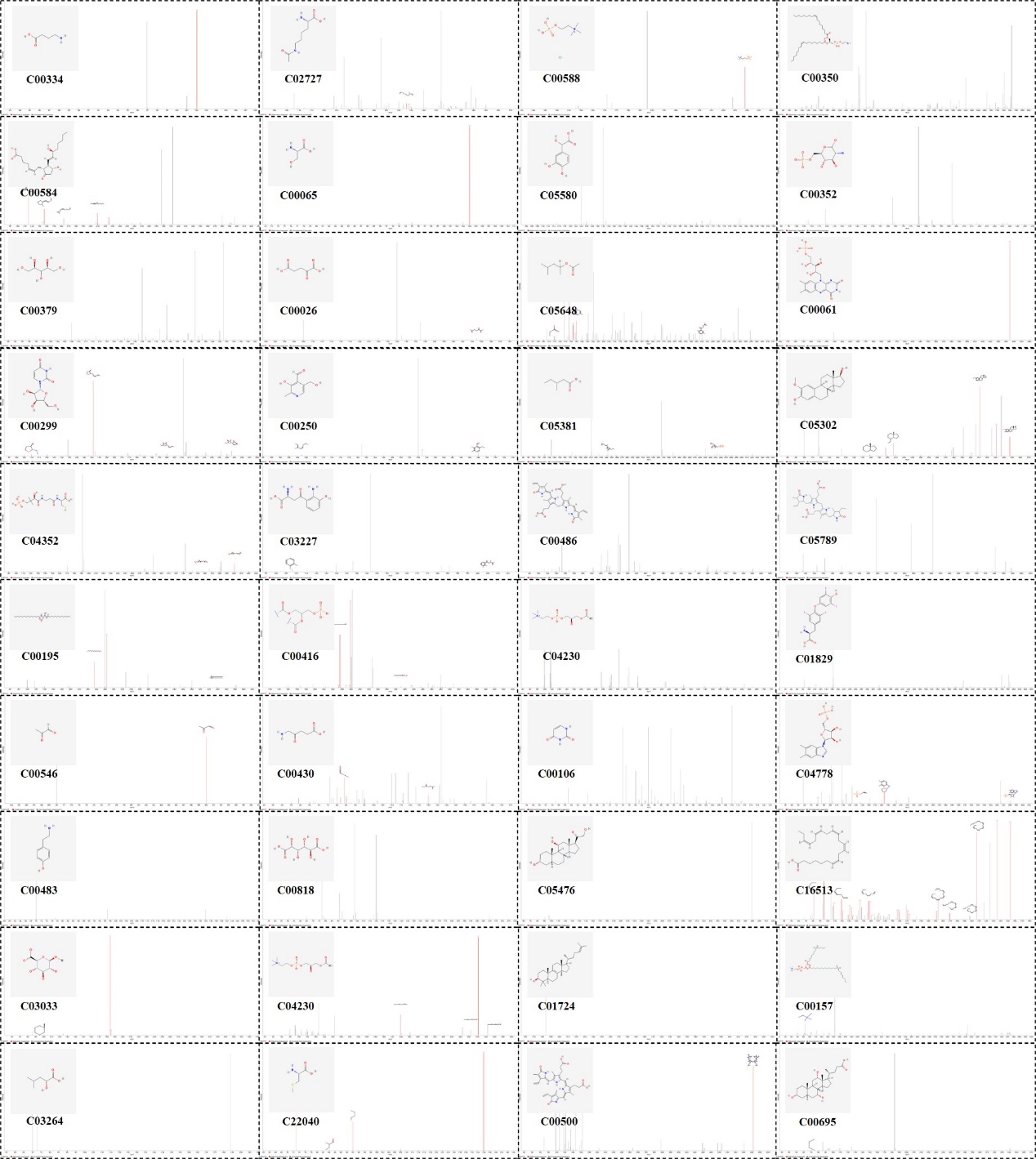


**Supplementary Figure 3** Secondary fragment ions characteristic maps of identified metabolites.


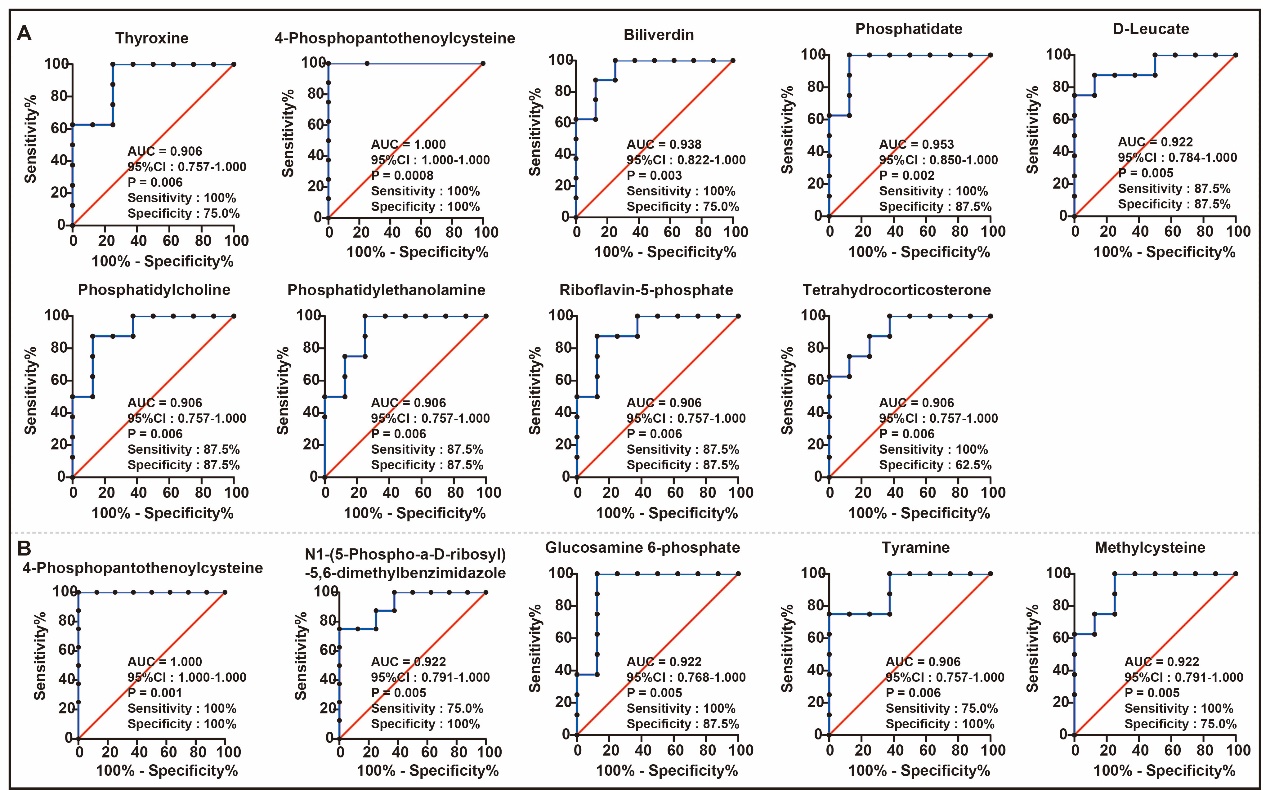


**Supplementary Figure 4** ROC analysis of metabolites. (A) the metabolites between the Yang_syn_+BGZ group and the Yang_syn_ group; (B) the metabolites between the Yin_syn_+BGZ group and the Yin_syn_ group.
